# Volume‒outcome relationships in bariatric surgery: a rapid review

**DOI:** 10.1038/s41366-025-01931-1

**Published:** 2025-10-24

**Authors:** Alessandro Campione, Ulrike Nimptsch, Helene Eckhardt, Cornelia Henschke

**Affiliations:** 1 Department of Health Care Management, Technische Universität Berlin, Berlin, Germany; 2 Institute of General Practice and Interprofessional Care, University Hospital Tübingen, Faculty of Medicine, Eberhard Karls Universität Tübingen, Tübingen, Germany

**Keywords:** Epidemiology, Health policy, Translational research

## Abstract

**Background:**

The treatment of obesity is complex and requires long-term multidisciplinary care. While behavioral, pharmacological and psychological therapies are integral, bariatric surgery remains the most effective intervention. Therapeutic success is influenced by factors such as comorbidities and potentially by the experience of the treatment facilities. This rapid review evaluates the evidence of volume-outcome associations in bariatric surgery, focusing on the endpoint of mortality.

**Methods:**

We performed a rapid review of the literature published after 2000, including adult patients (≥18 years) who underwent bariatric surgery for weight loss, or weight loss and diabetes management. Searches across EMBASE, MEDLINE, PubMed, and Cochrane Trials yielded 3540 records. The primary outcome was mortality; secondary outcomes included complications, morbidity and hospital stay. Mortality results were stratified by type of mortality and synthesized according to volume type (hospital or surgeon). Study quality was assessed using the ISPOR and ROBINS-E tools. An additional synthesis was conducted for studies above a quality score cutoff.

**Results:**

Thirty-six studies met the inclusion criteria. Of these, 12 studies examined the association between hospital volume and mortality, and five studies focused on surgeon volume. Eight of the hospital volume studies and four of the surgeon volume studies reported a positive association with reduced mortality; some showed mixed results. Most studies also linked higher volumes to fewer complications and shorter hospital stays. However, the focus was predominantly on hospital volume and short-term mortality, with limited evaluations of long-term outcomes or weight loss success. Overall study quality varied, with noted limitations including arbitrary volume thresholds.

**Conclusion:**

Higher hospital and surgeon volumes were associated with lower short-term mortality, fewer complications and shorter hospital stays. The association was more pronounced in higher-quality studies. Future research should aim to standardize volume definitions to improve comparability and support policy efforts to centralize care and enhance patient outcomes.

## Introduction

Obesity is a growing global health concern. As of 2022, one in eight people worldwide was living with obesity [1]. It is associated with a substantial loss of disability-free life years and increased premature mortality [2], estimated to reduce life expectancy by 2–4 years and to contribute to higher excess mortality than smoking [3]. The treatment management of obesity is complex and typically involves long-term multidisciplinary care across multiple medical disciplines and the integration of pharmacological and psychological therapies [4]. To date, bariatric surgery has emerged as the most effective therapeutic component for the treatment of obesity [5] and its associated diseases, such as diabetes type 2. While mortality rates are low [6], the effective treatment of obesity is complicated by its related comorbidities.

Postoperative complications occur in approximately 5–10% of patients and include bleeding, anastomotic leak, and infection [7]. Moreover, obesity itself is a risk factor for adverse short-term surgical events [8], and long-term postoperative mortality is mediated by accompanying comorbidities [9]. Several European countries—including Austria, Switzerland, Denmark, the United Kingdom and the Netherlands—have implemented minimum volume standards for bariatric surgery. Aiming to improve surgical outcomes and reduce complications and mortality, those standards range from 25 to 200 procedures a year and hospital, however with varying types of procedures involved [5, 10, 11]. These policy decisions were informed by studies reporting reduced mortality rates and complications when surgeries are performed by experienced surgeons and high-volume hospitals. For instance, a U.S. study of 14,716 patients reported lower odds ratios of hospital and short-term mortality in hospitals performing more than 100 procedures annually, compared to those performing fewer than 50 [12]. Similarly, another US study involving 14,714 patients from a retrospective registry identified a significant interaction between hospital and surgeon volume, further supporting the volume-outcome relationship, with high-volume hospitals defined as performing ≥300 procedures annually [13]. A large Scandinavian registry study of 49,977 bariatric surgeries reported improved composite hospital mortality and 90-day-reintervention rates for medium volumes (7–25 procedures/year), although no significant effect was observed for higher volumes [14].

Multiple studies with large sample sizes have also demonstrated significant associations between higher hospital volume and reduced perioperative morbidity, including lower rates of readmissions, reoperations, and specific complications [15–18]. Length of stay (LOS) was reported to be significantly shorter in high-volume hospitals and when surgeries were performed by high-volume surgeons [16, 19, 20].

While this volume-outcome relationship has been assessed in several studies, only a limited number of up-to-date systematic reviews are currently available. An umbrella review published in 2016 [21] summarizes three systematic reviews, including a health technology assessment from 2011 [22, 23] and 2012 [24], concluding that hospital and surgeon volume were inversely related to outcomes. However, these earlier works were partially focused on economic evaluation, did not capture the evidence published over the past decade and representing a gap in contemporary evidence, during which time both the volume and nature of bariatric procedures have evolved [25]. Additionally, in 2016 the International Federation for the Surgery of Obesity and Metabolic Disorders (ISFO) issued a joined statement recommending the consideration of weight loss surgery for the management of diabetes, thereby redefining the indications for bariatric procedures [26]. Since the introduction of laparoscopic techniques in 2000 [27], this review aims to systematically assess, update, and synthesize the evidence on the volume–outcome relationship in bariatric surgery generated over the past 23 years, thus addressing a gap in the current literature.

## Methods

We conducted a rapid review aiming to inform clinicians and health policy decision makers. This type of review was chosen against the backdrop of hospital reform in Germany, where hospital planning will be based on defined services in the future. Bariatric surgery is one of these defined services [28, 29]. We applied Preferred Reporting Items for Systematic Reviews and Meta-Analysis (PRISMA) 2020 [30], slightly adapted for rapid reviews based on the updated guidance on methods used in Cochrane rapid reviews methods guidance for rapid reviews of effectiveness [31]. The review was prospectively registered at PROSPERO platform (CRD42023398566) [32].

### Eligibility criteria

The following inclusion criteria were applied:

#### Population

Adult patients (≥18 years) undergoing invasive bariatric procedures for weight loss with or without diabetes. Stratification by indication was not performed, as available evidence suggests similar effects on mortality and life expectancy regardless of diabetes status [33]. Studies were excluded if more than 10% of the study population had malignant disease and the results were reported jointly for cancer and non-cancer patients. Studies with ≤10% of patients with malignancies were included even if data were not reported separately.

#### Intervention/exposure

The relative volume of invasive bariatric procedures was investigated at the hospital and surgeon level. This included the relative volume of high-volume surgeons compared to other surgeons or high-volume hospitals compared to hospitals with lower volumes of surgery procedures. Studies were excluded if more than 10% of the study population received other interventions and the results were only reported jointly. Studies with ≤10% of patients receiving other interventions were included even if data were not reported separately.

#### Context

Studies comparing at least two hospitals or surgeons in terms of bariatric surgery volume, explicitly distinguishing between high- and low-volume providers: (1) Low-volume hospitals vs. high-volume hospitals, and (2) Low surgeon caseload vs. high surgeon caseload.

#### Outcomes

Studies were included if they reported at least one of the following:

1. Mortality: all-cause mortality across any timeframe, including hospital mortality (perioperative, in-hospital mortality, in-hospital mortality among patients with complications (failure-to-rescue (FTR)), short-term (all cause 30-/90-day), and intermediate/long-term all-cause (1-/5-year) mortality.
2. Perioperative morbidity: adverse events of the intervention, re-intervention, re-admission and complications.
3. Disease-related morbidity: (long-term) weight/body mass index (BMI) reduction, comorbidity-related readmission rates (e.g., diabetes, arterial hypertension, sleep apnea).
4. Health-related quality of life (HRQoL): measured by validated instruments.
5. Length of stay (LOS) in hospital or intensive care units (ICU).

#### Study design

Eligible designs included randomized controlled trials (RCTs), controlled observational or interventional studies or trials (retrospective and prospective cohort studies). Systematic reviews were screened to identify relevant references.

#### Other

Only peer-reviewed publications from the year 2000 onward, in English or German were included. Full-text availability was required.

### Information sources and search strategy

A systematic literature search was conducted in PubMed via Pubmed, the Cochrane Library, EMBASE via Ovid and MEDLINE via Ovid on June 25, 2023. The search strategy was first developed in PubMed and subsequently adapted for the other databases. It combined controlled vocabulary—using Medical Subject Headings (MeSH) terms in PubMed/MEDLINE and the Cochrane Library, and Emtree terms in Embase—with free-text synonyms to capture hospital/surgeon volume (e.g., “Hospitals, High-Volume,” “surgical volume”), bariatric surgery (e.g., “Bariatric Surgery,” “gastric bypass”), obesity (e.g., “Obesity,” “overweight”), and diabetes (e.g., “Diabetes Mellitus,” “type 2 diabetes”). Logical operators, proximity searches, wildcards, and truncation were applied, with no restrictions on language or publication date. Exclusions were limited to non-human studies and non-research publication types (e.g., case reports, editorials). Full search details are provided in Appendix 1. Reference lists of eligible systematic reviews were cross-checked to ensure that all relevant studies were identified.

### Selection process

Following the methods of the Cochrane Rapid Reviews Methods Group, two reviewers (AC, CH) independently screened the titles and abstracts of a randomly selected 20% sample of search results using EndNote9.1. If agreement between reviewers was at least 90%, one reviewer screened the remaining records; otherwise, another 10% sample would have been screened until a 90% agreement was reached. One reviewer screened the remaining abstracts (AC). Full texts of all potentially relevant records were retrieved and screened by one reviewer (AC) of the research team against the review inclusion criteria. Any uncertainties in the review process were resolved through consultation with a second reviewer (CH). Studies excluded from full-text screening are found in Appendix 2.

### Data extraction and synthesis preparation

An Excel-based data extraction form was developed and piloted. One reviewer performed data extraction (AC). A second reviewer (CH) verified data accuracy. In case of uncertainties or missing data, the study authors were contacted via correspondence email. Discrepancies were solved through discussion.

Data items extracted:

- Basic study characteristics (e.g., country, study period, data source, sample size, surgery type, statistics and covariables).
- Volume definitions as main exposure of interest: (e.g., volume categories, case volume cutoffs or averages), as well as number of patients and number of units per case volume category.
- Outcomes and effect estimates (e.g., odds ratios (OR), risk ratios (RR), hazard ratios (HR), regression weights or percentages) and confidence intervals (CI) with the connected volume definition and reference categories, if reported. P-values were extracted for univariable results and in case CI were not reported.
- Model type (e.g., univariate or multivariate), with a focus on full models when multiple analyses were reported.
- Mortality outcomes were extracted and tabulated in three categories in accordance with PROSPERO protocol: (1) Hospital mortality; (2) short-term mortality; (3) intermediate/long-term mortality. Additional outcomes included (1) perioperative morbidity; (2) disease-related morbidity; (3) HRQoL; (4) LOS in hospital or ICU use.

### Synthesis method

We conducted a structured synthesis to summarize the findings of included studies according to the Synthesis Without Meta-analysis (SWiM) guideline [34]. We did not conduct a meta-analysis due to clinically and methodologically insufficiently homogenous studies, similarly to other systematic reviews [24]. Findings were synthesized by outcome type and stratified by volume category (hospital or surgeon or both). Mortality was the primary endpoint, and results were prioritized accordingly. Quality scores (see below) were used to rank studies, with high-quality studies appearing at the top of the summary tables. Separate analyses by mortality type were conducted to compare results from higher-quality studies according to their certainty and confidence.

### Risk of bias and reporting bias assessment

To evaluate the quality of included studies, the checklist of the International Society for Pharmacoeconomics and Outcome Research (ISPOR) was used [35]. The checklist contains 27 questions that focus on challenges specific to retrospective databases, disease registries and national survey data, considering the domains of the database, study methodology and study conclusions on the study level. Questions are scored as “Yes”, “Partially”, “No”, or “Not Applicable” (NA). As this instrument lacks a summary for the aggregated judgment measure, response counts for each study are listed next to their results. To evaluate risk of bias in the selection of the reported results, domain 7 of the ROBINS-E (Risk of Bias in Non-randomized Studies—of Exposure; Domain 7: Risk of bias in selection of the reported result) was applied for the exposure of being treated by surgeons or hospitals with their respective volumes [36]. This domain comprises five items on selective reporting with the response options “Yes”, “Probably Yes”, “Probably No”, “No”, or “No Information”. Item 7.1 (reporting in accordance with an available, pre-determined analysis plan) was uniformly assessed as “No Information” as none of the studies included such a plan. This is in accordance with the algorithms’ bias judgment, which assesses a study to “low risk”, “some concerns”, “high risk” or “very high” risk of reporting bias. The assessments were performed by one researcher (AC). Uncertainties were solved through discussion with a second researcher (CH).

To assess overall quality, the ISPOR checklist was appended with the ROBINS-E (domain 7) judgment, as the ISPOR checklist lacks a comprehensive appraisal of reporting quality. A composite Quality Score was calculated using an aggregate of ISPOR points and ROBINS-E Risk:

$$Quality\,Score={X}_{ISPOR\,\mathrm{'}\mathrm{'}Yes\mathrm{'}\mathrm{'}}+0.5* {X}_{ISPOR\,\mathrm{'}\mathrm{'}{\mathrm{Partially}}\mathrm{'}\mathrm{'}}+2* {{\mathbb{1}}}_{ROBINS\,\mathrm{'}\mathrm{'}Low\,Risk\mathrm{'}\mathrm{'}}$$

Outcomes were reported with both assessments and ranked within each volume-stratum (hospital or surgeon) based on the composite score to guide synthesis.

## Results

### Study selection

**Fig. 1:**
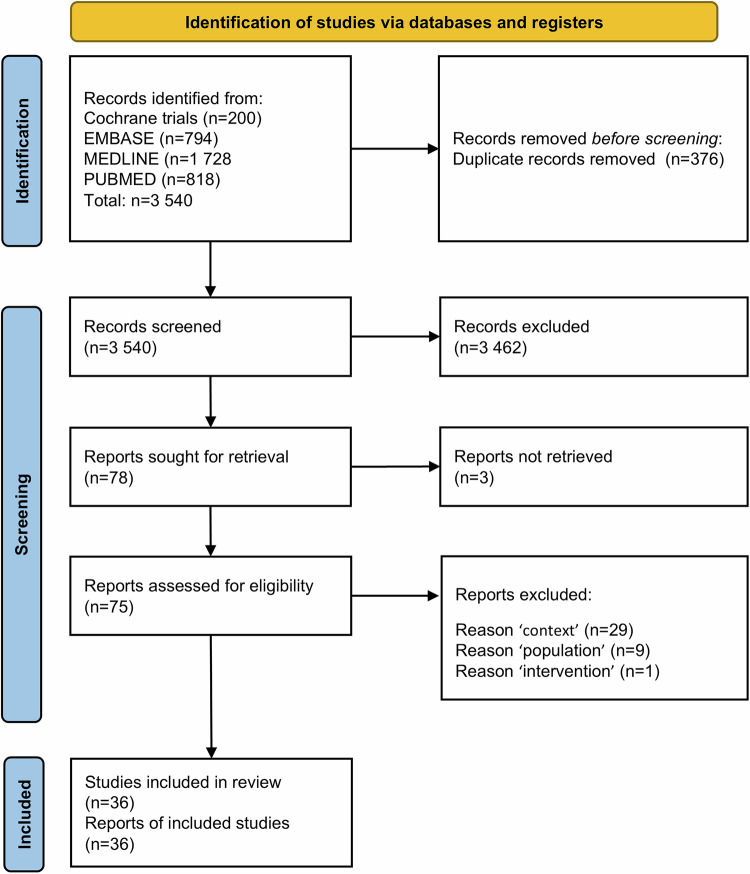
Flow chart of identified and included studies.

The literature extraction encompassed *n* = 3540 records for screening. After removal of duplicates and title/abstract screening, 75 titles were assessed with full-text screening, *n* = 36 publications met the inclusion criteria and were included in the review (Fig. 1). The most common reason for exclusion was wrong context.

Intervention sample sizes spanned between *n* = 933 and a maximum of *n* = 446,127. Among the included studies, 20 focused on hospital volume, eight on surgeon volume, and eight assessed both hospital and surgeon volume. Most studies came from the United States (69.4%), followed by Canada (8.3%). Other countries, including Denmark, Sweden, Finland, Italy, Australia, Taiwan and Brazil contributed single figures in terms of publications. Samples date back to the 1980s in two papers, while the most recent populations were sampled in 2018. All but two studies—both authored by the same first author—used retrospective databases or registries (the Longitudinal Assessment of Bariatric Surgery (LABS) clinical cohort from 2005–2007) [37, 38].

An overview of case volume categories, their case volume and corresponding units per case volume categories is provided in Appendix 3.

Table 1A–C shows the study characteristics by volume type, including the number of hospitals or surgeons involved (units), surgical indications and procedures, as well as statistics deployed for analysis and covariables.

**Table 1:** Characteristics of studies stratified by volume types.

Author, year of publication	Funding/declaration of conflict	Country (Region)	Study period	Study type/Data source	Intervention(s), incl. codes (Share, where available)	Surgical indication	Units (No. of surgeons)	Patients (*N*)	Statistics and covariables
**A. Characteristics of studies focusing on the relationship between surgeon volume and outcome**									
Altieri (2020) [20]	Study Funding: None. Conflict of Interest of Authors: Pryor: Speaker for Ethicon, Gore, Merck, Stryker, Medtronic, research support from Baronova and Obalon. Talamini: Consultant for Stryker. Spaniolas: Research support from Merck.	USA (New York)	2010–2014	Retrospective/ New York Statewide Planning and Research Cooperative System (SPARCS)	LRYGB (47.7%) or LSG (52.3%)	None reported, implied obesity	167	46,511 laparoscopic bariatric procedures	Generalized linear mixed model: sex, age, group, race, insurance, any comorbidity
Celio (2016) [63]	Study Funding: None declared. Conflict of Interest of Authors: None.	USA (North Carolina)	2011	Retrospective/ Bariatric Outcomes Longitudinal Database (BOLD)	SG (RYGB used for alternative volume)	None reported, implied obesity	736	16,547	Logistic regression: angina, asthma, CHF, DVT/PE, functional status impairment, hypertension, ischemic heart disease, hyperlipidemia, liver disease, obstructive sleep apnea, peripheral vascular disease, race, gender, glucose metabolism impairment
Celio (2017) [18]	Study Funding: None. Conflict of Interest of Authors: None.	USA (North Carolina)	2011	Retrospective/ Bariatric Outcomes Longitudinal Database (BOLD)	LRYGB	None reported, implied obesity	NR	32,521	Logistic regression: Patient demographics and comorbidities
Chao (2022) [53]	Study Funding: Public.Conflict of Interest of Authors: Chhabra: Consulting fees from Blue Cross Blue Shield of Massachusetts. Telem: Consulting fees from Medtronic. Dimick: Co-founder of ArborMetrix, Inc.	USA (Florida, Iowa, New York, Washington)	2013–2017	Retrospective/ Healthcare Cost and Utilization Project’s State Inpatient Databases (SID)	LRYGB (ICD-9-PCS: 44.38; ICD-10-PCS: 0D16479, 0D164K9, 0D164J9, 0D164Z9, 0D1647B, 0D164KB, 0164JB, 0D164ZB, 0D1647A, 0D164KA, 0D164JA, 0D164ZA, 0D1647L, 0D164KL, 0D164JL, 0D164ZL)	MSDRG code 619–621 for weight loss surgery	311	27,714	Multilevel logistic regression with surgeon clusters; Age, sex, race, Elixhauser comorbidities, year of surgery
Hunt (2020) [54]	Study Funding: None declared. Conflict of Interest of Authors: None.	Canada (Ontario)	2008–2015	Retrospective/ Canadian Institute for Health Information (CIHI)	RYGB (87.52%), SG (11.22%), and BPD (1.26%)	Patient identification through diagnosis morbid obesity and clarification via the procedure code for gastric bypass	29	13,836	Hierarchical logistic regression with hospital or surgeon clusters: Age, gender, comorbidities
Lopez (2002) [55]	Study Funding: None declared. Conflict of Interest of Authors: None declared.	USA (Florida)	1999	Retrospective/ State of Florida ARCA Database	Current Procedural Terminology codes for bariatric surgical procedures (NA)	(DRG) 278.01 (morbid obesity)	44	933	Chi-square tests; Wilcoxon rank sum tests; Student’s *t* tests: Univariable
Smith (2010) [37]	Study Funding: Public. Conflict of Interest of Authors: None declared.	USA (Pennsylvania, Columbia, New York, North Caroline, North Dakota, California, Washinton, Oregon)	2005–2007	Prospective observational/ Longitudinal Assessment of Bariatric Surgery (LABS)	RYGB	None reported, implied obesity	31	3410	Multilevel Poisson model clustering for surgeons: BMI, procedure type, 200 ft walk, history of DVA and OSA, years of experience
Smith (2013) [38]	Study Funding: Public Conflict of Interest of Authors: None declared.	USA (Pennsylvania, Columbia, New York, North Carolina, North Dakota, California, Washinton, Oregon)	2005–2007	Prospective observational/ Longitudinal assessment of Bariatric Surgery (LABS)	RYGB	None reported, implied obesity	31	3410	Log-linear model accounting for correlation between surgeon and site: Technical factors, BMI, history of DVT, OSA, ability to walk 200 ft
**B. Characteristics of studies focusing on the relationship between hospital volume and outcome**									
Carbonell (2005) [43]	Study Funding: None declared. Conflict of Interest of Authors: None.	USA	2000	Retrospective/ HCUP-NIS Database	ICD-9 CM gastric bypass (44.31, 44.32, 44.39)	Morbid obesity (278.01)	137	5876	Logistic regression: Morbidity: Age, sex, comorbidity, payer status, hospital bed size and region; Mortality: Sex, comorbidity, payer status, complications
Dimick(2009) [49]	Study Funding: Public. Conflict of Interest of Authors: None.	USA (NY)	2003–2006	Retrospective/ State Inpatient Database of New York	(ICD-9-CM primary procedure for gastric bypass (44.3, 44.31, 44.39), gastroplasty (44.69), laparoscopic gastric bypass (44.38), or laparoscopic gastric band placement (44.95) (NA)	Obesity (ICD-9-CM V854, 278.0, 278.00, 278.01, 278.1)	80	16,221	Hierarchical random effects model: risk adjusted morbidity
Encinosa (2009) [65]	Study Funding: None declared. Conflict of Interest of Authors: None. declared	USA	2001–2006	Retrospective/ MarketScan Commercial Claims and Encounter Database created by the Medstat Group, Inc.	Gastric banding or gastroplasty without gastric bypass; 43846 (RYGB); and 43845 and 43847 (other types of gastric bypass). 2005: introduction of laparoscopic bariatric CPT-4 codes for bypass: 43644 and 43645. Identification laparoscopic banding or gastroplasty without gastric bypass between 2005 and 2006: ICD-9-CM 4468 and 4495. Before 2005: Identification of laparoscopic bariatric surgeries by CPT-4 codes 43651–43659 (stomach laparoscopy) or 44200–44209 (intestinal laparoscopy). (Banding and gastroplasty without bypass 2001–2002: 5.47%; 2005-2006: 13.95%; gastric bypass 2001–2002: 94.53%; 2005–2006: 86.05%)	None reported, implied obesity	2001–2002: 308; 2005–2006: 532	Unclear	Logistic regression: Gastric banding without bypass, number of comorbidities, sex, age, insurance plan type, region, year, quarter of the year
Gould (2011) [44]	Study Funding: None declared. Conflict of Interest of Authors: None.	USA	2005–2007	Retrospective/ NIS from the Healthcare Cost and Utilization Project	ICD-9 CM: gastric bypass (44.3, 44.31, 44.39) (21%), laparoscopic gastric bypass (44.38) (58%), and laparoscopic adjustable gastric band (44.95) (21%)	Obesity (ICD-9-CM 278.0, 278.00, 278.01, 278.1)	137	22,509	Hierarchical GLM with hospital clusters: age, sex, procedure type, income, insurance, and all medical conditions
Hernandez-Boussard (2012) [15]	Study Funding: Public. Conflict of Interest of Authors: None.	USA	2005–2008	Retrospective/ NIS	ICD-9: RYGB (44.31, 44.39, 44.38) ICD-9-CM	Obesity diagnosis-related group (DRG) code 288	NR	354,478	Rao-Scott Chi-square test; Kruskal-Wallis test; Cochran-Armitage test: Risk adjusted for hospital cluster, age, sex, age sex interaction, DRG, comorbidities
Ibrahim (2017) [66]	Study Funding: Mainly public. (Conflict of Interest of Authors: Dimick: Financial interest in ArborMetrix. Dimick: Surgical Innovation Editor of JAMA Surgery.	USA (AR, AZ, FL, IA, MA, MD, NC, NE, NJ, NY, WA, WI)	2010–2013	Retrospective/ Healthcare Cost and Utilization Project’s State Inpatient Database	LRYGB (53.7%), open RYGB (3.8%), laparoscopic gastric band placement (11.6%), or laparoscopic SG (30.8%) (ICD-9-CM 43.89, 44.3, 44.31, 44.38, 44.39, 44.68, 44.95, 44.96, 44.97, 44.99, 44.5, 45.51, and 45.9)	Morbid obesity (ICD-9-CM codes 278.0, 278.00, and 278.01)	165	145,527	Hierarchical logistic regression with hospital clusters: Age, sex, race, Elixhauser comorbidities, operation type, year
Jafari (2013) [67]	Study Funding: None declared. Conflict of Interest of Authors: Nguyen: speaker bureau for GoreÒ	USA	2006–2010	Retrospective/ NIS	ICD-9: LRYGB (44.38) (97%), LSG (3%) (43.82 and 44.68)	Obesity and morbid obesity (278.0, 278.01, and 278.00)	NR	277,760	Logistic regression with robust standard errors: age, gender, ethnicity, hospital characteristics, comorbidities, procedural type
Kauppila (2020) [14]	Study Funding: Public and private non-profit. Conflict of Interest of Authors: None.	Denmark, Finland, Iceland, Norway and Sweden	1980–2012	Retrospective/ Nordic Obesity Surgery Cohort (NordOSCO)	Gastric bypass (73.4%); Vertical banded gastroplasty (11.0%); gastric banding (10.9%); other bariatric procedure (3.2%); malabsorptive procedure (1.6%)	None reported, implied obesity		49,977	Cox regression: Age, Sex, Charlson, type of surgery, surgical approach, year of surgery
Kohn (2010) [46]	Study Funding: Public. Conflict of Interest of Authors: None declared.	USA	1998–2006	Retrospective/ NIS	At least one of the following ICD-9-CM: 435, 436, 437, 4389, 4431, 4438, 4439, 445, 468, 4469, 4493, 4494, 4495, 4496, 4497, 4499, 4550, 4551, 4590, 4591 (gastric bypass, gastroplasty, malabsorptive, laparoscopic adjustable gastric band) (NA)	ICD-9-CM diagnosis codes: 2780, 27800, 27801, 27802	1045	102,069	Logistic regression with compound symmetric correlation for hospital clusters: CCI
Krell (2014) [39]	Study Funding: Public. Conflict of Interest of Authors: None declared.	USA (AZ, CA, FL, IA, MA, MD, NC, NE, NJ, NY, WA, WI)	2009–2010	Retrospective/ State Inpatient Databases	Laparoscopic or open bariatric surgical procedures, excluding laparoscopic adjustable gastric banding	None reported, implied obesity	198	31,240	Hierarchical modeling and empirical Bayes: age, sex, race, primary insurer, median ZIP code income, procedure type, Elixhauser comorbidities
Liu (2003) [68]	Study Funding: None declared. Conflict of Interest of Authors: None declared.	USA (CA)	1996–2000	Retrospective/ California’s Office of Statewide Health Planning and Development (OSHPD)	ICD-9: GB (44.31)	Obesity (ICD-9 codes 278.0x, 278.1, and 278.8)	101	16,232	Logistic regression: Age, race, sex, CCI
Markar (2023) [60]	Study Funding: Public. Conflict of Interest of Authors: None.	Sweden and Finland	1989–2020 in Sweden, 2018 in Finland	Retrospective/ Nordic Obesity Surgery Cohort (NordOSCo)	Bariatric surgery	Obesity diagnosis	NR	77,870 (68,084 Sweden; 9786 Finland)	Cox proportional model: Age, sex, country, CCI, type of bariatric surgical procedure
Morino (2007) [41]	Study Funding: None declared. Conflict of Interest of Authors: None.	Italy	1996–2006	Retrospective/ Italian Society of Obesity Surgery (SICOB)	ASGB (laparoscopic: 96.8%); VBG (laparoscopic: 41%); gastric bypasses (GBP) (laparoscopic: 86%); BPD (laparoscopic: 14%); biliointestinal bypasses; miscellaneous procedures	None reported, implied obesity	55 in 2005	13,431	Logistic regression: Sex, gender, BMI, hypertension, diabetes, hyperlipemia, surgical access, operative time, previous surgery, associated surgical procedures, type of procedure
Nguyen (2004) [47]	Study Funding: None declared. Conflict of Interest of Authors: None declared.	USA	1999–2002	Retrospective/ University HealthSystem Consortium (UHC)	ICD-9: RYGB (44.31, 44.39, and 44.3)	Obesity and morbid obesity (278.0, 278.01, 278.00, and 278.1) ICD-9	93	24,166	Pearson Chi-squared tests: Univariable
Pradarelli (2016) [59]	Study Funding: Public and private non-profit. Conflict of Interest of Authors: Dimick: Co-founder of ArborMetrix.	USA (MI)	2013– 2014	Retrospective/ Michigan Bariatric Surgery Collaborative (MBSC)	LSG	None reported, implied obesity	40	8693	Hierarchical mixed effect model with hospital clusters: age, gender, race, income level, insurance type, body mass index, smoking history, mobility limitations, comorbid conditions
Stenberg (2014) [51]	Study Funding: Public and private non-profit. Conflict of Interest of Authors: Hedenbro: Travel grants Covidien, Johnson & Johnson, grants Lund University funds, Crafoord foundation. Aslund: Grants Novo Nordisk, Stockholm County Council, Diabetes Theme enter at Karolinska Institutet, Stockholm. Sundbom: Expert testimony the Swedish National Board of Health and Welfare, royalties from Studentlitteratur for books for medical students. Aslund: Grants the Swedish government used to run the Scandinavian Obesity Surgery Registry.	Sweden	2007– 2012	Retrospective/ Scandinavian Obesity Surgery Registry (SOReg)	LGB	None reported, implied obesity	44 (unclear)	25,038	Logistic regression: Patient-specific risk factors
Svarts (2022) [16]	Study Funding: None. Conflict of Interest of Authors: None.	Sweden	2007– 2016	Retrospective/ Scandinavian Obesity Surgery Registry (SOReg)	Gastric bypass (90%) and gastric sleeve (10%)	None reported, implied obesity	51	52,703	Hierarchical models with hospital clusters: Logistic: Complications; Poisson model: LOS: Age, comorbidities, sex, surgery characteristics
Tsui (2020) [17]	Study Funding: None declared. Conflict of Interest of Authors: Spaniolas: Grant from Merck and Advisory Board for Mallickrodt. Pryor: Honoraria for speaking for Ethicon, Medtronic, Stryker, and Gore and is a consultant for Merck, Obalon, and Baronova.	USA (NY)	2006–2012	Retrospective/ New York Statewide Planning and Research Cooperative System (SPARCS)	ICD-9: SG (43.82, 43.89)	43,775 with primary diagnosis of 278.00-02)	NR	8389	Cox proportional model: age, blood loss anemia, hemorrhage, fluid and electrolyte disorder, vascular disease
Varban (2015) [48]	Study Funding: Public.Conflict of Interest of Authors: None declared.	USA (AZ, CA, FL, IA, MA, MD, NC, NE, NJ, NY, WA, WI)	2006–2011	Retrospective/ State Inpatient Databases (SID)	ICD-9 CM: LAGB, RYGB (44.95 and 44.38, respectively) (NA)	Morbid obesity (ICD-9-CM 278.0, 278.00, 278.01, V77.8) and surgery (DRG 288 until October 1, 2007 and MSDRG 619–621 after October 1, 2007)	NR	446,127 overall	Logistic regression: Age, race, sex, type of insurance, Elixhauser comorbidities
Wilson (2015) [69]	Study Funding: None declared. Conflict of Interest of Authors: None.	USA	2009–2011	Retrospective/ University Health Consortium (UHC) Clinical Database/ Resource Manager (CDB/RM)	ICD-9: Gastric bypass (44.3, 44.31, 44.38, 44.39) (colectomy, lung resection, and aortic surgery)	None reported, implied obesity	UHC consists of 118 academic medical centers and 298 affiliated hospitals	256,694 overall, GB: 62,010	Logistic regression: Gender, race, severity of illness, discharge status, LOS, age
**C. Characteristics of studies focusing on the relationship between both hospital and surgeon volume, and outcome**									
Bouchard (2020) [42]	Study Funding: Private non-profit. Conflict of Interest of Authors: None.	Canada (Quebec)	2006– 2012	Retrospective/ RAMQ and MED-ECHO	RYGB (68.7%) and SG (31.3%) (278.0, 278.9)	Obesity (E66.01, E66.2)	Surgeons: 34; Hospitals: 15	2623	Multilevel models accounting for hospital or surgeon and patient cluster: Age, sex, SES, CCI, year
Chadwick (2023) [19]	Study Funding: Public and private. Conflict of Interest of Authors: Caterson:Advisory board of InsideOut (for eating disorders), the board of Obesity Australia, and Executive Management Committee of the Australian and New Zealand Bariatric Surgical Register. Formerly president of the World Obesity Federation.	Australia	2015– 2020	Retrospective/ Australia and New Zealand Bariatric Surgery registry (ANZBSR)	SG (84.5%;), RYGB (5.5%), OAGB (5.2%) and LAGB (4.8%)	None reported, implied obesity	Hospitals: 104; Surgeons: 212	63,604	Linear regression: Patient demographic variables, procedure type, operation status (primary or conversion), defined adverse event type
Chiu (2012) [70]	Study Funding: None. Conflict of Interest of Authors: None.	Taiwan	1997–2008	Retrospective/ Taiwan Bureau of National Health Insurance (BNHI)	ICD-9-CM: Gastric bypass, incl. MGB, (44.31) and RYGB (44.39), laparoscopic gastric bypass (including laparoscopic MGB), and LRYGB (44.38), open gastroplasty (including VBG) (44.69), or laparoscopic gastroplasty (incl. LVBG) (44.68) and LAGB (44.95) Open gastric bypass = 392 (14.66%); laparoscopic gastric bypass = 834 (31.19%); open vertical banded gastroplasty = 1042 (38.97%); laparoscopic vertical banded gastroplasty = 406 (15.18%)	Obesity (ICD-0-CM code 278.00, 278.01, 278.02, or 278.1)	Hospitals: 278; Surgeons: 435	2674	Hierarchical linear model with hospital clusters (not surgeon clusters): age, gender, CCI, and bariatric procedure
Doumouras (2017) [50]	Study Funding: None. Conflict of Interest of Authors: None.	Canada (Ontario)	2009–2015	Retrospective/ Ontario Bariatric Network, CIHI	Gastric bypass (88.1%) and SG (11.9%)	Purpose of weight loss	Hospitals: 9; Surgeons: 29	13,256	Hierarchical regression: Morbidity; hierarchical logistic regression: Mortality: gender, age, comorbidities, annual hospital volume, annual surgeon volume, procedure, fellowship teaching center status, year of procedure
Hollenbeak (2008) [12]	Study Funding: None declared. Conflict of Interest of Authors: None declared.	USA (PA)	1999–2003	Retrospective/ Health Care Cost Containment Council (PHC4)	ICD-9: Gastroenterostomy without gastrectomy (44.3), high gastric bypass (44.31) and other gastroenterostomy (44.39) (NA)	Morbid obesity, incl. 278.0 (overweight and obesity), 278.01 (morbid obesity), 278.00 (obesity, unspecified), and 278.1 (localized adiposity), and to discharge with DRG 288 (operating room procedures obesity)	Hospitals: 1999: 119; 2001: 168; 2003: 114 | Surgeons: NR	14,716 procedures	Logistic regression: Mortality; generalized model with gamma distribution: LOS: age, gender, race, MedisGroup severity, payor type
Murr (2007) [40]	Study Funding: Partly private. Conflict of Interest of Authors: None declared.	USA (FL)	1999–2003	Retrospective/ Florida Agency of Health Care Administration Hospital Discharge Database	ICD-9: high gastric bypass (44.31) and gastroenterostomy (44.39) (NA)	ICD-9: Morbid obesity (278.01)	Hospitals: 93; Surgeons: 97-197	19,174	Logistic regression: Year, age, gender
Torrente (2013) [13]	Study Funding: None declared. Conflict of Interest of Authors: None declared.	USA (PA)	1999–2003	Retrospective/ Pennsylvania Health Care Cost Containment Council	44.3 (gastroenterostomy without gastrectomy), 44.31 (high gastric bypass), and 44.39 (other gastroenterostomy) ICD-9 (NA)	ICD-9: Morbid obesity, incl. 278.0 (overweight and obesity), 278.00 (obesity, unspecified), 278.01 (morbid obesity), and 278.1 (localized adiposity), and discharge with a DRG 288 (operating room procedures for obesity)	NR	14,714	Logistic regression: Mean age, gender, race, ASG score, payer type
Kelles (2009) [45]	Study Funding: None declared. Conflict of Interest of Authors: None.	Brazil	2004–2007	Retrospective/ Brazilian Unified Health System (SUS)	RYGB	None reported, implied obesity	Hospitals: 16; Surgeons: 48	2167	Logistic regression: mortality: BMI, age; LOS: Age, hypertension, BMI

*ASGB* adjustable silicone gastric banding, *ASG* accident severity grade, *BPD* biliopancreatic diversion, *CCI* Charlson comorbidity index, *CPT* current procedural terminology, *CIHI* Canadian Institute for Health Information, *CHF* chronic heart failure, *DVA* developmental venous anomaly, *DVT* deep vein thrombosis, *DRG* diagnosis-related group code, *ISPOR* The International Society for Pharmacoeconomics and Outcomes Research, *LABS* longitudinal assessment of bariatric surgery, *LAGB* laparoscopic adjustable gastric band, *LRYGB* laparoscopic Roux-en-Y gastric bypass, *LVBG* laparoscopic vertical banded gastroplasty, *LOS* length of stay, *MED-ECHO* Ministry of Health’s Maintenance et Exploitation des Donnees pour l’Etude de la Clientele Hospitaliere, *NA* not available, *MGB* mini gastric bypass, *NIS* Healthcare Research and Quality Nationwide Inpatient Sample, *OAGB* one-anastomosis gastric bypass, *OSA* obstructive sleep apnea, *RAMQ* Regie de l’Assurance Maladie du Quebec, *ROBINS-E* the risk of bias in non-randomized studies–of exposure, *RYGB* Roux-en-Y gastric bypass, *SG* sleeve gastrectomy, *SES* socioeconomic status, *PE* pulmonary embolism, *VBG* vertical banded gastroplasty, *AR* Arkansas, *AZ* Arizona, *FL* Florida, *IA* Iowa, *MA* Massachusetts, *MD* Maryland, *NC* North Carolina, *NE* Nebraska, *NJ* New Jersey, *NY* New York, *PA* Pennsylvania, *WA* Washington, *WI* Wisconsin.

“None declared” means that no conflict-of-interest statement was explicitly provided in the publication. “None” indicates that the authors explicitly declared the absence of conflicts of interest or funding.

### Association between hospital/surgeon volume and mortality, incl. quality appraisal

In total, 17 studies reported mortality outcomes. Results were stratified by volume type and tabulated with their ISPOR and ROBINS-E score. Studies that analyzed both hospital and surgeon volume appear twice in the tables and are marked with an asterisk (*). Based on inference-based findings (i.e., reporting confidence intervals or *p* values), Tables 2 and 3 show a total of 33 effect estimates, 23 related to hospital volume, seven related to surgeon volume, and one study addressing the interaction between surgeon and hospital volume for two mortality outcomes appears in both tables [13]. Two studies presented non-inference-based results, which were therefore excluded from the quantitative synthesis [39, 40]. Among the 12 hospital volume studies with inferential analysis, eight found significant associations between higher volume and lower mortality, while two studies reported mixed results [41, 42]. Regarding surgeon volume, four out of five studies found a significant volume-mortality association. One study, which assessed combined mortality and morbidity, reported mixed findings [42]. One study was excluded from the synthesis because it employed an interaction term between hospital and surgeon volume that could not be clearly assigned to either category. However, that study reports significant associations between hospital and short-term mortality for the interactions of surgeon and hospital volumes [13].

For studies analyzing hospital volume, mixed effects were found in an earlier 2007 study [41]. Reported results were stratified by procedures, with significant hospital-volume effects observed for overall short-term mortality and vertical-banded gastroplasty (VBG) procedures, but not for other procedures. However, the inference was based on univariable tests of percentages and lacks adjustment for confounding variables. A similar limitation was noted in a 2005 study, which did not find a statistically significant effect [43]. A more recent large population-based registry study found a significant effect, but the exposure pattern was inconsistent: medium-volume hospitals were associated with significantly lower mortality compared to low-volume hospitals, while high-volume hospitals showed no significant difference [14]. Two studies reported composite outcomes including death, with the former contributing mixed effects by different, surgery-specific volume definitions [42, 44]. Although two other older studies did not report any inferential statistics, one reported a lower percentage of hospital mortality in higher-volume hospitals, while the other observed no meaningful variation in mortality percentages across volume levels [39, 40].

**Table 2:** Hospital volume effects.

Author, year of publication	Hospital mortality: all cause perioperative mortality, in-hospital mortality, in-hospital mortality among patients with complications (failure to rescue) Short term mortality: all cause 30-/90-day mortality Intermediate/long-term mortality: all cause 1-/5-year mortality	Quality appraisal	
		ISPOR (Yes/ Partially/No/NA)	ROBINS-E (Domain 7^b^)
Bouchard^a^ (2020) [42]	**SG: 90-day major morbidity** (aOR, 1 unit = 10 cases): 0.99 (0.97–1.01) **RYGB: 90-day major morbidity** (aOR, 1 unit = 10 cases): 0.86 (0.77–0.96) {Included: complications: bleeding, venous thromboembolic event, pneumonia, macrovascular events (myocardial infarction and strokes), postoperative infection/leak, shock, need for de novo hemodialysis, reintubation, prolonged intubation (>48 h), prolonged LOS ≥ 7 days), or ***mortality***}	(10/8/2/7)	Very High
Torrente^a^ (2013) [13]	**Hospital mortality:** (aOR, interaction of surgeon x hospital volumes): Low x Low vs. Low x Medium: 0.75 (0.25–2.30); Low x High: 0.57 (0.07–4.39); High x Low: 1.08 (0.51–2.30); High x Medium: 0.39 (0.17–0.89); High x High: 0.2 (0.07–0.56) | **Short-term mortality:** (aOR, interaction of surgeon x hospital volumes): Low x Low vs. Low x Medium: 0.64 (0.26–1.59); Low x High: 0.63 (0.15–2.7); High x Low: 0.89 (0.49–1.64); High x Medium: 0.5 (0.28–0.9); High x High: 0.3 (0.15–0.6)	(11/2/7/7)	Low
Kauppila (2020) [14]	**Composite hospital mortality and reintervention:** (90 days, aHR): Lowest vs. Medium: 0.7 (0.52–0.95); Highest: 0.8 (0.61–1.07)	(12/4/4/7)	High
Gould (2011) [44]	**Occurrence of one or more severe post- operative in-hospital complication and in-hospital mortality (aOR):** > 25 vs. 1–24: 1.13 (0.84–1.52); ≥50 vs. 1–49: 1.17 (0.94–1.45); ≥75 vs. 1–74: 1.21 (0.92–1.36): ≥100 vs. 1–99: 1.22 (1.01–1.49); ≥125 vs. 1–124: 1.31 (1.1–1.67); ≥150 vs. 1–149: 1.36 (1.1–1.67); ≥175 vs. 1–174: 1.57 (1.27–1.94); ≥200 vs. 1–199: 1.61 (1.29–2.01)	(11/2/7/7)	Very High
Markar (2023) [60]	**Intermediate/long-term mortality:** (10-year, aHR): Q1 (lowest) vs. Q2: 0.88 (0.81–0.96); Q3: 0.87 (0.78–0.97); Q4: 0.82 (0.73–0.93) (highest)| Continuous: 0.99 (0.99–1.00)	(12/0/8/7)	Low
Hollenbeak^a^ (2008) [12]	**Hospital mortality**: (aOR): Hospitals: High vs. Medium: 2.42, *p* = 0.18; Low: 2.34, *p* = 0.016| **Short-term mortality**: (aOR): Hospitals: High vs. Medium: 2.07, *p* = 0.12; Low: 2.01, *p* = 0.01	(9/5/6/7)	Low
Krell (2014) [39]	**Hospital mortality:** (Mean risk-and reliability adjusted rate, % (range)): Low: 0.1% (0.1%–0.1%); Medium: 0.1% (0.1%–2.4%); High: 0.1% (0.1%–0.1%)	(10/2/8/7)	Low
Kohn (2010) [46]	**Hospital mortality:** (aOR, incremental): 0.99717 (0.99579–0.99856)	(11/3/6/7)	High
Nguyen (2004) [47]	**Hospital mortality:** (Observed to expected, %): Low: 3.9% vs. High: 1.2%, *p* < 0.05; Medium: 1.7%	(11/2/7/7)	High
Jafari (2013) [67]	**Hospital mortality**: (aOR): High vs. Low: 2.5 (1.3–4.8)	(11/2/7/7)	High
Murr^a^ (2007) [40]	**Hospital mortality:** (Mean ± sd %): 1–9: 2.87% ± 11.0%; 10–99: 0.62% ± 1.9%; 100–199: 0.12% ± 0.3%; 200–499: 0.22% ± 0.3%; ≥500: 0.25% ± 0.3%	(8/3/9/7)	High
Carbonell (2005) [43]	**Hospital mortality:** (percentage): Very low: 0.54%; Low: 0.95%; Medium: 0.87%; High: 0.1%, *p* = 0.06	(8/2/10/7)	Very High
Varban (2015) [48]	**Hospital mortality:** (aOR, LAGB volume): 2006–2007: >125 vs. 50–125: 1.63 (0.31–8.51); <50: 2.40 (0.65–8.81); 2008–2009: >125 vs. 50–125: 0.34 (0.09–1.32); <50: 1.72 (0.74–4.02); 2010–2011: NR (aOR, LRYGB volume): 2006–2007: >125 vs. 50–125: 1.67 (0.73–3.81); <50:0.95 (0.31–2.91); 2008–2009: >125 vs. 50–125: 0.71 (0.29–1.73); <50: 1.39 (0.61–3.15); 2010–2011: >125 vs. 50–125: 0.79 (0.31–2.02); <50: 1.13 (0.43–2.97);	(8/2/10/7)	High
Hernandez-Boussard (2012) [15]	**Hospital mortality:** (Risk-adjusted, percentage): Low: 3.02%; Mid: 2.98%; High: 2.45%, *p* < 0.0001| **Failure to rescue:** (Risk adjusted, rate per 1000): Low: 160.41; Mid: 118.78; High: 101.7, *p* < 0.0001	(5/3/12/7)	High
Morino (2007) [41]	**Short-term mortality** (60 days, percentages): Overall: Low: 0.51% vs. High: 0.19%, *p* < 0.01; BPD: Low: 0.83% vs. High: 0.79%, *p* = nonsignificant; GBP: Low: 0.89% vs. High: 0.45%, *p* = nonsignificant; VBG: Low: 0.58% vs. High: 0.1%, *p* < 0.05; ASGB: Low: 0.19% vs. High: 0.08%, *p* = nonsignificant	(6/1/11/7)	High

*aHR* adjusted hazard ratio, *aOR* adjusted odds ratio, *aRR* adjusted risk ratio, *ASGB* adjustable silicone gastric banding, *BPD* biliopancreatic diversion, *GBP* gastric bypass surgery, *ISPOR* The International Society For Pharmacoeconomics and Outcomes Research, *LABS* longitudinal assessment of bariatric surgery, *LAGB* laparoscopic adjustable gastric band, *LOS* length of stay, *LRYGB* laparoscopic Roux-en-Y gastric bypass, *NA* not applicable, *NR* not reported, *OAGB* one-anastomosis gastric bypass, *OS* overall survival, *ROBINS-E* the risk of bias in non-randomized studies–of exposure, *RYGB* Roux-en-Y gastric bypass, *sd* standard deviation, *SG* sleeve gastrectomy, *VBG* vertical banded gastroplasty.

^a^Studies that analyzed both hospital and surgeon volume appear twice in the tables (Tables 2 and 3).

^b^Domain 7 = Risk of bias in selection of reported results.

**Table 3:** Surgeon volume effects.

Author, year of publication	Hospital mortality: all cause perioperative mortality, hospital mortality, hospital mortality among patients with complications (failure to rescue) Short term mortality: all cause 30-/90-day mortality Intermediate/long-term mortality: all cause 1-/5-year mortality	Quality appraisal	
		ISPOR (Yes/ Partially/No/NA)	ROBINS-E (Domain 7^c^)
Bouchard^b^ (2020) [42]	**SG: 90-day major morbidity** (aOR, 1 unit = 10 cases): Surgeons: 0.99 (0.93–1.06) **RYGB: 90-day major morbidity** (aOR, 1 unit = 10 cases): Surgeons: 0.82 (0.71–0.94) {Included: complications: bleeding, venous thromboembolic event, pneumonia, macrovascular events (myocardial infarction and strokes), postoperative infection/leak, shock, need for de novo hemodialysis, reintubation, prolonged intubation ( > 48 h), prolonged length of stay (LOS ≥ 7 days), or ***mortality***}	(10/8/2/7)	Very High
Torrente^b^ (2013) [13]	**Hospital mortality:** (aOR, interaction of surgeon x hospital volumes): Low x Low vs. Low x Medium: 0.75 (0.25–2.30); Low x High: 0.57 (0.07–4.39); High x Low: 1.08 (0.51–2.30); High x Medium: 0.39 (0.17–0.89); High x High: 0.2 (0.07–0.56) | **Short-Term mortality:** (aOR, interaction of surgeon x hospital volumes): Low x Low vs. Low x Medium: 0.64 (0.26–1.59); Low x High: 0.63 (0.15–2.7); High x Low: 0.89 (0.49–1.64); High x Medium: 0.5 (0.28–0.9); High x High: 0.3 (0.15–0.6)	(11/2/7/7)	Low
Hollenbeak^b^ (2008) [12]	**Hospital mortality:** (aOR): High vs. Medium: 2.65, *p* = 0.019; Low: 3.59, *p* = 0.003| **Short-term mortality:** (aOR): High vs. Medium: 1.61, *p* = 0.091; Low: 2.27, *p* = 0.004	(9/5/6/7)	Low
Murr^b^ (2007) [40]	**Hospital mortality**: (Mean ± sd %): 1–5: 1.32% ± 10.5%; 6–99: 0.83% ± 2.6%; 100–199: 0.27% ± 0.5%; 200–499: 0.28% ± 0.3%; ≥500: 0.22% ± 0.4%	(8/3/9/7)	High
Smith (2010) [37]	**Composite event:** (**Death**/DVT/PE/no discharge within 30 days/post-bariatric surgery operation/post-bariatric surgery re-admission): (aRR): 0–24 vs. 25–49: 0.61 (0.30–1.25); 50–99: 0.41 (0.22–0.79); >100: 0.35 (0.16–0.76)	(6/3/11/7)	High
Smith (2013) [38]	**Composite event from smith 2010:** (**Death/**DVT/PE/no discharge within 30 days/post-bariatric surgery operation/post-bariatric surgery re-admission): (aRR): Surgeon’s LABS volume (per 10 cases/year): 0.93 (0.88–0.98)	(5/3/12/7)	High
Kelles^a^ (2009) [45]	**Short-term mortality:** (aOR): High vs. Low: 6.211 (1.9–20.26)	(5/3/12/7)	High

*aHR* adjusted hazard ratio, *aOR* adjusted odds ratio, *aRR* adjusted risk ratio, *DVT* deep vein thrombosis, *ISPOR* the International Society for Pharmacoeconomics and Outcomes Research, *LABS* longitudinal assessment of bariatric surgery, *LOS* length of stay, *NA* not applicable, *PE* pulmonary embolism, *ROBINS-E* the risk of bias in non-randomized studies–of exposure, *RYGB* Roux-en-Y gastric bypass, *sd* standard deviation, *SG* sleeve gastrectomy.

^a^Intended to analyze hospital volume but excluded in modeling.

^b^Studies that analyzed both hospital and surgeon volume appear twice in the tables (Tables 2 and 3).

^c^Domain 7 = Risk of bias in selection of reported results.

Studies analyzing surgeon volume partly overlapped with those on hospital volume. Two publications by the same author used composite endpoints including death, both demonstrating significant volume-outcome effects [37, 38]. These studies were based on the same sample but applied different volume definitions. In addition to these, several studies that showed significant hospital volume effects also reported significant surgeon volume associations [12, 13]. The only study rejecting an association found non-significant effects for 90-day major composite mortality and morbidity following sleeve gastrectomy (SG), for both surgeon and hospital volume [42]. An older 2009 study initially planned to assess both hospital and surgeon volume, but later excluded hospital volume from modeling process [45]; therefore, it is included only under surgeon volume results.

### Mortality by mortality type

**Fig. 2:**
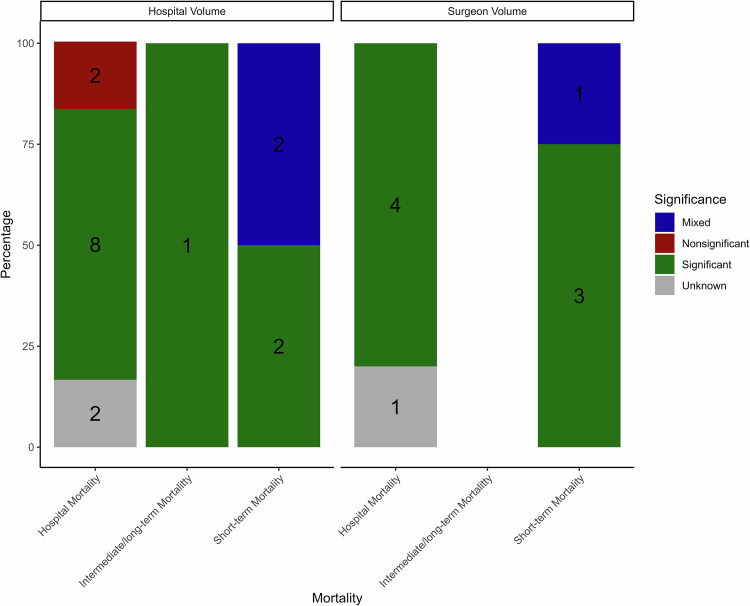
Study conclusions stratified by mortality outcome.

Figure 2 summarizes study results by type of mortality outcome—categorized as in-hospital, short-term, or intermediate/long-term mortality—and by overall study conclusion (significant, non-significant, mixed, or unknown when no inference was available). Studies were grouped by hospital volume and surgeon volume. This additional counting method was produced to decrease the effect of multiple reported results (here as “mixed”) and provide a more balanced overview on the evidence. In total, two studies reported no significant association between hospital volume and in-hospital mortality. In contrast, eight studies identified a positive relationship, while two studies lacked inferential statistics and were categorized as unknown. Only one study measured intermediate/long-term mortality in relation to hospital volume. Mixed results were found in three studies: two examining short-term mortality using hospital volume, and one using surgeon volume. Overall, the evidence tends to support a significant volume-outcome relationship. As a sensitivity analysis, studies with unknown effects may conservatively be treated as non-significant to assess the robustness of overall conclusions.

### High-quality studies

After isolating high-quality studies based on predefined quality scoring criteria, a total of three surgeon volume and nine hospital volume entries remained. Notably, all three studies in the surgeon volume category also included analyses of hospital volume. Among the nine hospital volume studies, one showed mixed results [42], one did not demonstrate a clear exposure-outcome relationship [14], and one lacked inferential analysis and was limited to descriptive statistics [39]. Overall, restricting the synthesis to higher-quality studies as defined in this review supports a consistent volume-mortality relationship, indicating that higher provider volume is associated with better mortality outcomes. This is further corroborated by restricting the analysis to studies employing adjusted models, with those studies exceeding the quality score cutoff delineated by a bold line (Appendix 4).

### Hospital and surgeon volume: outcome associations and quality appraisal

Perioperative morbidity was significantly related to hospital volume in ten studies. Six studies reported mixed effects, while two studies reported no association. Mixed effects were usually related to analysis of different complications (e.g., specific vs. overall), re-operations or some differing volume definitions [17, 20, 39, 46–48]. Two studies lacked a clear exposure pattern, one of which also failed to demonstrate such a pattern for mortality outcomes [14, 39]. A large U.S. study including 12 states (2014) found significant associations for overall complications, but none for reoperation [39]. A 2004 study focusing on Roux-En-Y gastric bypass (RYGB) procedures reported significant effects for overall complications, and single complications such as pulmonary, medical, wound complications, and readmission, but not for pneumonia, thrombosis or embolisms, and postprocedural hemorrhage [47]. The study with the largest sample size, also from the U.S., found significant associations between volume and serious complications for both laparoscopic adjustable gastric banding (LAGB) and LRYGB volume. However, significant volume–reoperation associations were only found for LAGB in the time-blocks of 2006–2007 and 2008–2009, but not in 2010–2011 [48]. All four studies using disease-related morbidity found a significant association with hospital volume [16, 43, 49, 50]. However, only one of them examined specific morbidity outcomes (total weight loss after 1 year) [16]. LOS was significantly associated with hospital volume in seven studies [12, 16, 19, 43, 47, 51, 52], while one study did not find a significant association [15], and another provided non-interpretable results [45].

For surgeon volume, three studies found a significant association with perioperative morbidity. The other three reported mixed results. A recent 2021 study using a large U.S. database of RYGB procedures found significant improvement in complications when using aggregate complications, but none of the single complications resulted in significance [53]. Only one study using a large sample from Canada analyzed overall disease-related morbidity in relation to surgeon volume, reporting a significant association [54]. All six studies assessing LOS in relation to surgeon volume found significant associations [12, 19, 20, 45, 52, 55].

No studies in either volume category used validated quality of life measures. A comprehensive overview of additional outcomes and quality appraisal details is provided in Appendix 5. Furthermore, Appendix 4 provides an overview of studies using adjusted models across all outcome domains.

## Quality assessment of included studies

The median quality score across all studies, as described in Section “Risk of bias and reporting bias assessment”, was 11.5 points. A total of 17 studies exceeded this cutoff and were classified as “higher quality”, including nine studies addressing mortality-related outcomes. Assessment of reporting quality using ROBINS-E (domain 7) demonstrated variability. Three studies were identified with a very high risk of reporting bias. Twenty-two studies were assessed at high risk of reporting bias. In contrast, 11 studies were evaluated as having a low risk of bias. Study quality, including reporting quality, suffered from a lack of justification or validation of exposure definitions. Only few studies comprehensively reported the numeric volume ranges or cutoffs, contextualized these with specific nomenclature (e.g., “high”; “middle” or “low”), and provided justifying citations, sensitivity analyses or explanation for their choices such as data- or context-driven approaches to defining volume categories. Notably, several ISPOR quality assessment items emerged as critical constraints on study quality. These included the description of reliability, validity and quality checks, the rationale for study design selection, dealing with censoring and missing data, explanation of statistical modeling approaches, discussion of influential cases, testing of statistical assumptions, dealing with multiple tests and model prediction assessment.

## Discussion

This rapid review provides updated evidence on the volume-outcome relationship in bariatric surgery for adults indicated for weight loss with or without diabetes. Our results suggest better outcomes—specifically lower mortality and complication rates—when surgery is performed at centers and/or by surgeons with higher volumes. This correlation aligns with the “practice makes perfect” hypothesis, which suggests that increased procedural volume enhances technical skills, fosters greater familiarity with complex conditions, and improves the management of complications [56]. Furthermore, high-volume hospitals are more likely to have access to superior resources and infrastructure, as they are more often located in urban areas and tend to attract more referrals and patient visits—factors that, in turn, further improve outcomes [57]. Remarkably, none of the studies included assessed quality of life as an outcome. Consequently, the body of evidence can map outcomes such as mortality and complications but remains partially blind to patient-reported outcomes that are relevant to the therapeutic goals of multidisciplinary obesity management, highlighting a gap in the evaluation of patient-centered measures. Future research may address this gap by employing validated instruments, such as the EQ-5D-5L for overall health related quality of life, SF-36 or obesity surgery-specific instruments like the Bariatric Analysis and Reporting Outcome System (BAROS), which assess weight loss, comorbidities, and quality of life [58].

The review also points to methodological concerns in the studies included. Some studies evaluated mortality outcomes using aggregate measures that included mortality among other endpoints [37, 38, 42, 44]. While this aggregation increases statistical power, it diminishes the ability to discern which specific outcomes are influenced by surgical volume. Nevertheless, this issue was limited to a subset of studies, and the overall conclusions remained robust when these were excluded. Moreover, most studies primarily focused on short-term or hospital-based mortality, with limited exploration of intermediate or long-term mortality. Similarly, disease-related morbidity was assessed less frequently compared to perioperative morbidity. The predominant focus on short-term outcomes likely reflects data availability and incomplete follow-up in registries but restricts insights into volume-outcome relationships to certain outcome domains. Among adjusted studies, those using morbidity as an outcome reported positive associations with hospital volume in both categorical and continuous analyses. Lower-quality studies more frequently yielded mixed results, typically due to some complications lacking statistical significance. These studies more often assessed surgeon volume, while the highest quality study analyzing morbidity reported significant associations with surgeon volume of LRYGB and sleeve gastrectomy (LSG) [20]. Only a few studies on disease-related morbidity met the high-quality threshold; one of such study used a composite of mortality and various complications, showing statistically significant associations for hospital volumes of SG procedures only [42]. Two lower-quality studies, both below the quality threshold, examining the association between disease-related morbidity and surgeon volume, reported one significant [54] and one non-significant association [50] for different but overlapping procedures. Across both lower and higher-quality studies, LOS was significantly associated with surgical and hospital volume, whether analyzed incrementally or categorically. It was also observed that mortality studies were generally older compared to those assessing complications or morbidity, indicating a shift in research focus over time. Despite some overlap with older systematic reviews [22–24], this rapid review includes more recent studies of higher quality, providing updated insights into the volume-outcome relationship.

One notable limitation of this review is the lack of standardized volume categorizations across studies. Many reports did not employ literature-based or data-driven categorizations of volumes, complicating the interpretation of volume-outcome correlations. This aspect contributed to a downgrade in quality during the appraisal. Certain studies defined volume according to policy-relevant criteria, such as the Bariatric Surgery Center of Excellence criteria recommendations [13, 19, 54, 59], applied changepoint analysis [20], or performed sensitivity analyses [60]. Analyses restricted to lower volumes may fail to detect effects that manifest only beyond a certain threshold, while high cutoffs can exceed the point of diminishing marginal returns in the volume-outcome association, as shown in one included study [20]. However, this rapid review observed evidence in favor of higher volumes across various volume definitions, including different forms of marginal analyses (Appendix 4). Studies with higher quality were more likely to use data-driven or policy-oriented approaches and transparently report volume thresholds, positively influencing their assessment under ROBINS-E criteria. Approaches not identified in the included literature encompass spline models, receiver-operator curves, and chi-square automatic interaction detection algorithms, as exemplified in a nationwide 2019 study of digestive cancer surgery [61]. While these data-driven methods can detect cutoffs, their generalizability may be limited when applied to the same dataset used for effect estimation.

The absence of causal frameworks, such as propensity score matching methods, in the included studies restricts their findings to associative results. Furthermore, this analysis is not equipped to recommend specific cutoff points for minimum volume standards, nor to synthesize the magnitude or clinical significance of effects. The heterogeneity in volume categorization, procedures, adjustment variables, and statistical modeling approaches across studies complicates estimation of these sources of variation. Future research should aim to quantify clinical benefit and identify policy-relevant volume thresholds using meta-analytic approaches.

Some studies employed limited covariables [40] and basic descriptive analyses [47, 55], while others used extensive adjustment for patient risk factors, facility characteristics or surgeon-specific variables, and morbidity-related variables, even accounting for time, cohort or cluster effects in their models [16, 39, 59]. However, even studies deploying a broader range of adjustments may be subject to residual confounding, as micro-level patient characteristics are often not captured in registries and hence remain unaccounted for. While the quality appraisal based on ISPOR checklist combined with ROBINS-E instrument contains assessments of data source reliability, validity, and reporting, the underreporting of adverse events, such as complications or morbidity, cannot be excluded. Items receiving negative assessments often concerned data quality checks, the discussion of influential cases, and the handling of censoring, in addition to other statistical considerations. Accordingly, the distribution of acceptable and lower study quality may restrict the quality of synthesized evidence and the conclusions of this review. In a synthesis of higher-quality studies, we found acceptable quality to sustain the conclusions. Yet, this review lacked an appraisal of publication bias, such that the skew towards positive results may stem from a lack of published null results. Additionally, the quality score was defined based on practical considerations, with the aim of enabling within-study comparisons. The score has not been validated, and the applied quality threshold is solely anchored in the quality points assigned within this review. Assessments for both instruments are reported for each study.

Geographically, the majority of the populations analyzed originated from the United States, with fewer studies representing non-Western, educated, industrial, rich and democratic (WEIRD) countries. This distribution reflects the higher prevalence of obesity in WEIRD countries but limits the generalizability of our results to these geographical regions. European health systems, for example, differ significantly in terms of access, cost structures, and the organization of bariatric surgery [62]. Broadening the scope of research to include more diverse healthcare contexts would improve the external validity of these findings.

We also found some overlaps in the data sources used, particularly with population-based registries. For instance, two reports using the Scandinavian Obesity Surgery Registry included overlapping observation periods, although the newer report extended the analysis by four additional years and differed in volume definitions [16, 51]. Similarly, a study from 2020 analyzed a Nordic obesity cohort [14], which was extended by up to 8 years in a 2023 publication that analyzed different outcomes [60]. Two 2016 and 2017 studies by the same author inquired the Bariatric Outcomes Longitudinal Database, but were included as they differed in analyzed interventions [18, 63]. The most meaningful overlap was observed in two studies by the same first author, using the Longitudinal Assessment of Bariatric Surgery (LABS) database, with the later 2013 study differing only in additional outcomes and volume definitions [37, 38].

Although studies using US-based registries and databases occasionally demonstrated overlap, they often differed in methodological designs, outcomes analyzed, type of surgery and regions of patient population. Such overlaps were carefully considered during data synthesis to prevent redundancy, as substantial overlap can lead to inflated study results without contributing new information. However, none of the studies exhibited complete overlaps without distinguishing features in methodology or procedures analyzed.

The findings of this rapid review are comparable with previous literature, including a systematic review from 2012 that analyzed volume-outcome relationships in bariatric surgery. That review, which included studies up to 2011, reported similar conclusions regarding improved outcomes with higher procedural volumes [24]. Although this systematic review did not explicitly exclude studies on surgical indications for malignancies, it found both strong evidence supporting volume-outcome associations and generally acceptable study quality, as assessed by the Newcastle-Ottawa Quality Assessment scale. In contrast, our review identified a trend towards higher-quality studies in more recent publications, suggesting improvements in methodological rigor over time.

Significant limitations of this study relate to methodological limitations inherent to rapid reviews. Only one reviewer screened all titles and extracted data. Although other reviewers verified data accuracy and were consulted in case of uncertainty, the risk of single-reviewer bias in screening and quality assessments may occur [64]. Furthermore, database coverage was limited, and synthesis depth was restricted by both heterogeneity and time constraints. The review process faced additional challenges related to the data extraction of results. Multiple studies lacked comprehensive reporting on volume definitions and intervention characteristics, which led to quality downgrades during assessment. Variability in reporting surgical indications and diagnostic codes further complicated study selection, necessitating inferred interpretation based on outcomes and study aims. To address reporting discrepancies, ROBINS-E domain 7 was included in the quality assessment. Moreover, some studies presented high-density results through multiple model variations. In these cases, priority was given to fully adjusted models that included volume as a key variable, ensuring that synthesized results reflected the most methodologically sound evidence. Given the multidimensional nature of obesity, the study underlines the importance of considering bundled care models that centralize expertise and resources in high-volume hospitals. Such a model could enhance consistency in outcomes and optimize the quality of care delivered. Notably, few studies in the review analyzed success metrics beyond mortality and complication rates, such as quality of life, BMI reduction, diabetes management, or other morbidity-related outcomes. This observation may stem from the widespread acceptance of bariatric surgery as an effective intervention, making further scrutiny of its impact on these parameters seem redundant, as previously evidenced in meta-analyses focusing on morbidity outcomes [48].

In conclusion, the studies included in this review indicate a relationship between hospital and surgeon volume and patient outcomes. For hospital volume, most reports identified an association with short-term mortality. As highlighted in the existing literature, there is a predominant focus on short-term outcomes, while robust evidence supporting a volume-outcome relationship for long-term mortality and morbidity remains unsubstantiated in this review. Given that long-term mortality rates following bariatric surgery are generally low, this tendency may adequately capture the relevant mortality risks in the context of bariatric surgery. Similarly, perioperative and postoperative complications were more frequently analyzed and demonstrated improvements with increasing procedural volumes.

The methodological quality of included studies varied, reflecting inconsistent reporting and a lack of standardized volume definitions. Future research should prioritize the substantiation of volume definitions, including the provision of relevant citations or the application of data-driven methodologies to delineate which volume thresholds qualify as “high” or “low.” A more rigorous and transparent validation of volume cutoffs would not only enhance the comparability of studies but also support the generalizability of findings. This, in turn, could inform evidence-based policy decisions on the centralization of bariatric care to optimize patient outcomes. Furthermore, to enhance comparability across studies, researchers investigating volume-outcome effects across different medical disciplines should converge on standardized reference categories for volume levels in modeling and inference. Such standardization would facilitate more consistent evidence synthesis and policy recommendations. Additionally, prospective cohort studies incorporating validated HRQoL instruments could address the current gap regarding possible volume-outcome associations with long-term therapeutic benefits and quality of life improvements. The strong heterogeneity noted in this and other reviews may be mitigated through studies based on international registries and harmonized procedural definitions, while future reviews could account for this variability by applying meta-analytic methods to estimate the clinical significance of reductions in mortality or complications across volume definitions. Similarly, primary studies using causal frameworks could provide more robust estimates of clinical benefit, thereby avoiding the limitations inherent to purely associative analyses.

## Supplementary information

Appendix

## Data Availability

The data extracted in this work are available in the publication, its appendices, and from the corresponding author on reasonable request.
