# Supplementary material for: Volume‒outcome relationships in bariatric surgery: a rapid review
Source: Int J Obes (Lond). 2025 Oct 24;50(1):33–52. doi: 10.1038/s41366-025-01931-1 (PMC12855007; doi:10.1038/s41366-025-01931-1)
Supplement: Supplementary file 1 — Appendix [file 41366_2025_1931_MOESM1_ESM.docx]

**Appendix**

**Appendix 1: Search strings**

Cochrane Trials:

| #1 | MeSH descriptor: [Hospitals, High-Volume] explode all trees |
| --- | --- |
| #2 | MeSH descriptor: [Hospitals, Low-Volume] explode all trees |
| #3 | ((case NEXT volume*) OR (service NEXT volume*)):ab,kw,ti |
| #4 | (minim* NEAR/2 (volume OR volumes OR caseload OR workload OR "case-load" OR "work-load")):ab,kw,ti |
| #5 | ((high-volume* OR low-volume* OR caseload* OR volume OR volumes OR workload OR case-load OR work-load OR small OR large OR type OR size) NEAR/2 (hospital OR hospitals OR clinic OR clinics OR facility OR facilities OR center OR centers OR centre OR centres OR provider* OR institution*)):ab,kw,ti |
| #6 | ((high-volume* OR low-volume* OR caseload* OR volume OR volumes OR workload OR "case-load" OR "work-load") NEAR/2 (surg* OR patient* OR procedure*)):ab,kw,ti |
| #7 | ((surgeon NEXT experience*) OR (surgical NEXT experience*) OR (surgery NEXT experience*) OR (surgeon NEXT performance) OR (surgical NEXT performance) OR (surgery NEXT performance)):ab,kw,ti |
| #8 | ((centrali* OR decentrali* OR regionali*) NEAR/2 (volume OR volumes OR caseload* OR workload* OR "case-load" OR "work-load" OR hospital* OR treat* OR service*)):ab,kw,ti |
| #9 | MeSH descriptor: [Surgeons] explode all trees |
| #10 | (surgeon* OR physician* OR specialist*):ab,kw,ti |
| #11 | (caseload* OR volume OR volumes OR workload OR "case-load" OR "work-load"):ab,kw,ti |
| #12 | #9 OR #10 |
| #13 | #12 AND #11 |
| #14 | ((hospital OR hospitals OR clinic OR clinics OR facility OR facilities OR center OR centers OR centre OR centres OR provider* OR surgeon* OR physician* OR provider* OR specialist* OR volume OR volumes) NEAR/2 outcome*):ab,kw,ti |
| #15 | ((small OR large OR type OR size) NEAR/2 (hospital OR hospitals OR clinic OR clinics OR center OR centers OR centre OR centres OR institution)):ab,kw,ti |
| #16 | {OR #1-#8, #13-#15} |
| #17 | MeSH descriptor: [Gastric Bypass] explode all trees |
| #18 | MeSH descriptor: [Gastroplasty] explode all trees |
| #19 | MeSH descriptor: [Anastomosis, Roux-en-Y] explode all trees |
| #20 | MeSH descriptor: [Jejunoileal Bypass] explode all trees |
| #21 | MeSH descriptor: [Gastroenterostomy] explode all trees |
| #22 | MeSH descriptor: [Biliopancreatic Diversion] explode all trees |
| #23 | MeSH descriptor: [Digestive System Surgical Procedures] this term only |
| #24 | MeSH descriptor: [Gastrectomy] explode all trees |
| #25 | MeSH descriptor: [Fundoplication] explode all trees |
| #26 | MeSH descriptor: [Biliary Tract Surgical Procedures] this term only |
| #27 | MeSH descriptor: [Pyloromyotomy] explode all trees |
| #28 | MeSH descriptor: [Gastropexy] explode all trees |
| #29 | MeSH descriptor: [Gastrostomy] explode all trees |
| #30 | ((jejunoileal OR "jejuno-ilial" OR (jejuno NEXT ilial) OR gastric OR anastomosis OR anastom* OR duoden* OR "Roux-Y" OR "omega-loop" OR omega) NEAR/2 (bypass OR bypass*)):ab,kw,ti |
| #31 | ("roux-en-Y" OR LRYGB* OR LAGB OR gastrectomy OR gastroplasty OR gastrogastrostomy OR "gastro-gastrostomy" OR gastroenterostomy):ab,kw,ti |
| #32 | ((bypass OR bypass* OR gastrointestinal OR malabsorpti* OR gastric OR "restrictive") NEAR/2 (surg* OR operation OR operations OR intervention OR interventions)):ab,kw,ti |
| #33 | ((gastric OR stomach) NEAR/2 (stapl* OR plication OR imbrication OR balloon)):ab,kw,ti |
| #34 | ((gastrointestinal OR biliopancreatic) NEAR/2 diversion*):ab,kw,ti |
| #35 | ((lap OR silicon OR gastr*) NEAR/2 (band* OR sleeve*)):ab,kw,ti |
| #36 | ((mason* OR malabsorpti*) NEAR/2 procedure*):ab,kw,ti |
| #37 | (switch* NEAR/2 duoden*):ab,kw,ti |
| #38 | ((gastro* OR gastric OR stomach OR duoden*) NEAR/2 (surg* OR resect* OR operation OR operations OR remov*)):ab,kw,ti |
| #39 | {OR #17-#38} |
| #40 | MeSH descriptor: [Body Weight Changes] this term only |
| #41 | MeSH descriptor: [Weight Loss] explode all trees |
| #42 | MeSH descriptor: [Overweight] explode all trees |
| #43 | MeSH descriptor: [Obesity] explode all trees |
| #44 | MeSH descriptor: [Overnutrition] explode all trees |
| #45 | MeSH descriptor: [Body Weight] this term only |
| #46 | MeSH descriptor: [Obesity, Abdominal] explode all trees |
| #47 | MeSH descriptor: [Diabetes Mellitus] this term only |
| #48 | MeSH descriptor: [Diabetes Mellitus, Type 1] this term only |
| #49 | MeSH descriptor: [Diabetes Mellitus, Type 2] this term only |
| #50 | MeSH descriptor: [Metabolic Syndrome] explode all trees |
| #51 | MeSH descriptor: [Nutrition Disorders] this term only |
| #52 | MeSH descriptor: [Gastroparesis] explode all trees |
| #53 | MeSH descriptor: [Glucose Metabolism Disorders] explode all trees |
| #54 | ((obes* OR overweight OR (over NEXT weight) OR weightloss OR "weight-loss" OR (weight NEXT los*) OR "body-weight" OR bodyweight OR (weight NEXT reduction) OR (weight NEXT red*) OR BMI OR (body NEXT mass NEXT index))):ab,kw,ti |
| #55 | (diabetes* OR diabetic* OR gastropare* OR (metabolic NEXT disorder)):ab,kw,ti |
| #56 | {OR #40-#55} |
| #57 | #39 AND #56 |
| #58 | MeSH descriptor: [Bariatric Surgery] explode all trees |
| #59 | ((bariatric OR obes* OR antiobes* OR (anti NEXT obes*) OR (weight NEXT reduc*) OR metabolic) NEAR/2 (surg* OR operation OR operations OR resect*)):ab,kw,ti |
| #60 | ((bariatric NEXT procedur*) OR (bariatric NEXT intervention*)):ab,kw,ti |
| #61 | ((gastric OR stomach OR gastro*) NEAR/2 (stimulat* OR electro* OR electrostimulat* OR electro-stimulat* OR electric* OR neurostimulat* OR neuromudulat* OR pacemak* OR pace-make* OR Medtronic OR Transcend* OR (Abiliti NEXT System*) OR (Exilis NEXT system*) OR TANTALUS-DIAMOND* OR TANTALUS* OR MetaCure* OR Enterra*)):ab,kw,ti |
| #62 | MeSH descriptor: [Electric Stimulation Therapy] this term only |
| #63 | (gastric* OR gastro*):ab,kw,ti |
| #64 | #62 AND #63 |
| #65 | {OR #58-#61, #64} |
| #66 | #57 OR #65 |
| #67 | #16 AND #66 |

PubMed:

| #1 | "Hospitals, High-Volume"[Mesh] OR "Hospitals, Low-Volume"[Mesh] |
| --- | --- |
| #2 | "high-volume hospitals"[tiab: ~3] OR "low-volume hospitals"[tiab: ~3] OR "high-volume centers"[tiab: ~3] OR "high-volume centres"[tiab: ~3] OR "low-volume centers"[tiab: ~3] OR "low-volume centres"[tiab: ~3] OR "high-volume clinics"[tiab: ~3] OR "low-volume clinics"[tiab: ~3] OR "high-volume facilities"[tiab: ~3] OR "low-volume facilities"[tiab: ~3] OR "hospital volumes"[tiab: ~3] OR "clinic volumes"[tiab: ~3] OR "center volumes"[tiab: ~3] OR "centre volumes"[tiab: ~3] OR "facility volumes"[tiab: ~3] OR "patient volumes"[tiab: ~3] OR "provider volumes"[tiab: ~3] OR "surgical volumes"[tiab: ~3] OR "procedure volumes"[tiab: ~3] OR "procedural volumes"[tiab: ~3] OR "treatment volumes"[tiab: ~3] |
| #3 | "high-volume hospital"[tiab: ~3] OR "low-volume hospital"[tiab: ~3] OR "high-volume center"[tiab: ~3] OR "high-volume centre"[tiab: ~3] OR "low-volume center"[tiab: ~3] OR "low-volume centre"[tiab: ~3] OR "high-volume clinic"[tiab: ~3] OR "low-volume clinic"[tiab: ~3] OR "high-volume facility"[tiab: ~3] OR "low-volume facility"[tiab: ~3] OR "hospital volume"[tiab: ~3] OR "clinic volume"[tiab: ~3] OR "center volume"[tiab: ~3] OR "centre volume"[tiab: ~3] OR "facility volume"[tiab: ~3] OR "patient volume"[tiab: ~3] OR "provider volume"[tiab: ~3] OR "surgical volume"[tiab: ~3] OR "procedure volume"[tiab: ~3] OR "procedural volume"[tiab: ~3] OR "treatment volume"[tiab: ~3] |
| #4 | ("high-volume*"[tiab] OR "low-volume*"[tiab] OR caseload[tiab] OR volume[tiab] OR volumes[tiab] OR workload[tiab] OR "case-load"[tiab] OR "work-load"[tiab] OR small[tiab] OR large[tiab] OR type[tiab] OR size[tiab]) AND (provider*[tiab] OR institution*[tiab]) |
| #5 | "surgeon experience*"[tiab] OR "surgical experience*"[tiab] OR "surgery experience*"[tiab] OR "surgeon performance"[tiab] OR "surgical performance"[tiab] OR "surgery performance"[tiab] |
| #6 | "centralisation hospital"[tiab:~3] OR "centralization hospital"[tiab:~3] OR "centralisation service"[tiab:~3] OR "centralization service"[tiab:~3] OR "centralisation hospitals"[tiab:~3] OR "centralization hospitals"[tiab:~3] OR "centralisation services"[tiab:~3] OR "centralization services"[tiab:~3] OR "centralisation treatment"[tiab:~3] OR "centralization treatment"[tiab:~3] OR "centralisation treatments"[tiab:~3] OR "centralization treatments"[tiab:~3] |
| #7 | "Surgeons"[Mesh] |
| #8 | (surgeon*[tiab] OR physician*[tiab] OR specialist*[tiab]) |
| #9 | #7 OR #8 |
| #10 | caseload[tiab] OR volume[tiab] OR volumes[tiab] OR workload[tiab] OR "case-load"[tiab] OR "work-load"[tiab] |
| #11 | #9 AND #10 |
| #12 | "case volume*"[tiab] OR "service volume*"[tiab] OR "minimum volume*"[tiab] OR "minimal volume*"[tiab] OR "minimum caseload*"[tiab] OR "minimum workload*"[tiab] OR "minimal workload*"[tiab] |
| #13 | "volume outcome"[tiab:~3] OR "volume outcomes"[tiab:~3] OR "volumes outcome"[tiab:~3] OR "volumes outcomes"[tiab:~3] |
| #14 | (small[tiab] OR large[tiab]) AND (hospital[tiab] OR hospitals[tiab] OR clinic[tiab] OR clinics[tiab] OR center[tiab] OR centers[tiab] OR centre[tiab] OR centres[tiab]) |
| #15 | "hospital size*"[tiab] OR "clinic size*"[tiab] OR "center size*"[tiab] OR "centre size*"[tiab] OR "case volume*"[tiab] OR "service volume*"[tiab] |
| #16 | "hospital type"[tiab:~3] OR "clinic type"[tiab:~3] OR "institution type"[tiab:~3] OR "hospitals type"[tiab:~3] OR "clinics type"[tiab:~3] OR "institutions type"[tiab:~3] OR "center type"[tiab:~3] OR "centre type"[tiab:~3] OR "centers type"[tiab:~3] OR "centres type"[tiab:~3] |
| #17 | #1 OR #2 OR #3 OR #4 OR #5 OR #6 OR #11 OR #12 OR #13 OR #14 OR #15 OR #16 |
| #18 | "Gastric Bypass"[MeSH Terms] OR "Gastroplasty"[MeSH Terms] OR "Jejunoileal Bypass"[MeSH Terms] OR "Gastroenterostomy"[MeSH Terms] OR "anastomosis, roux en y"[MeSH Terms] OR "Biliopancreatic Diversion"[MeSH Terms] OR "Digestive System Surgical Procedures"[Mesh:NoExp] OR "Gastrectomy"[Mesh] OR "Fundoplication"[Mesh] OR "Biliary Tract Surgical Procedures"[Mesh:NoExp] OR "Pyloromyotomy"[Mesh] OR "Gastropexy"[Mesh] |
| #19 | "Gastrostomy"[Mesh:NoExp] |
| #20 | (jejunoileal[tiab] OR "jejuno-ilial"[tiab] OR "jejuno ilial"[tiab] OR gastric[tiab] OR anastomosis[tiab] OR anastom*[tiab] OR duoden*[tiab] OR "Roux-Y"[tiab] OR "omega-loop"[tiab] OR omega[tiab]) AND (bypass[tiab] OR bypass*[tiab]) |
| #21 | "roux-en-Y"[tiab] OR LRYGB*[tiab] OR LAGB[tiab] OR gastrectomy[tiab] OR gastroplasty[tiab] OR gastrogastrostomy[tiab] OR "gastro-gastrostomy"[tiab] OR gastroenterostomy[tiab] |
| #22 | (bypass[tiab] OR bypass*[tiab] OR gastrointestinal[tiab] OR malabsorpti*[tiab] OR gastric[tiab] OR "restrictive"[tiab]) AND (surg*[tiab] OR operation[tiab] OR operations[tiab] OR intervention[tiab] OR interventions[tiab]) |
| #23 | (gastric[tiab] OR stomach[tiab]) AND (stapl*[tiab] OR plication[tiab] OR imbrication[tiab] OR balloon[tiab]) |
| #24 | (gastrointestinal[tiab] OR biliopancreatic[tiab]) AND diversion*[tiab] |
| #25 | (lap[tiab] OR silicon[tiab] OR gastr*[tiab]) AND (band*[tiab] OR sleeve*[tiab]) |
| #26 | (mason*[tiab] OR malabsorpti*[tiab]) AND procedure*[tiab] |
| #27 | switch*[tiab] AND duoden*[tiab] |
| #28 | (gastro*[tiab] OR gastric[tiab] OR stomach[tiab] OR duoden*[tiab]) AND (surg*[tiab] OR resect*[tiab] OR operation[tiab] OR operations[tiab] OR remov*[tiab]) |
| #29 | #18 OR #19 OR #20 OR #21 OR #22 OR #23 OR #24 OR #25 OR #26 OR #27 OR #28 |
| #30 | "Body Weight Changes"[Mesh:NoExp] OR "Overweight"[MeSH Terms] OR "Obesity"[MeSH Terms] OR "Overnutrition"[MeSH Terms] OR "Body Weight"[MeSH Terms] OR "Weight Loss"[MeSH Terms] OR "Obesity, Abdominal"[Mesh] |
| #31 | "Diabetes Mellitus"[Mesh:NoExp] OR "Diabetes Mellitus, Type 1"[Mesh] OR "Diabetes Mellitus, Type 2"[Mesh:NoExp] OR "Diabetes Mellitus, Type 1"[Mesh:NoExp] OR "Metabolic Syndrome"[Mesh] OR "Nutrition Disorders"[Mesh:NoExp] OR "Overnutrition"[Mesh] |
| #32 | (obes*[tiab] OR overweight[TIAB] OR "over weight"[tiab] OR weightloss[tiab] OR "weight-loss"[tiab] OR "weight los*"[tiab] OR "body-weight"[tiab] OR "bodyweight"[tiab] OR "weight reduction"[tiab] OR "weight red*"[tiab] OR BMI[tiab] OR "body mass index"[tiab]) |
| #33 | "Gastroparesis"[Mesh] OR "Glucose Metabolism Disorders"[Mesh] |
| #34 | diabetes*[tiab] OR diabetic*[tiab] OR gastropare*[tiab] OR "metabolic disorder"[tiab] |
| #35 | #30 OR #31 OR #32 OR #33 OR #34 |
| #36 | #29 AND #35 |
| #37 | "Bariatric Surgery"[Mesh] |
| #38 | (bariatric[tiab] OR obes*[tiab] OR antiobes*[tiab] OR "anti obes*"[tiab] OR "weight reduc*"[tiab] OR metabolic[tiab]) AND (surg*[tiab] OR operation[tiab] OR operations[tiab] OR resect*[tiab]) |
| #39 | "bariatric procedur*"[tiab] OR "bariatric intervention*"[tiab] |
| #40 | (gastric[tiab] OR stomach[tiab] OR gastro*) AND (stimulat*[tiab] OR electro*[tiab] OR electrostimulat*[tiab] OR "electro-stimulat*"[tiab] OR electric*[tiab] OR neurostimulat*[tiab] OR neuromudulat*[tiab] OR pacemak*[tiab] OR pace-make*[tiab] OR Medtronic[tiab] OR Transcend*[tiab] OR "Abiliti System*"[tiab] OR "Exilis system*"[tiab] OR "TANTALUS-DIAMOND*"[tiab] OR "TANTALUS*"[tiab] OR MetaCure*[tiab] OR Enterra*[tiab]) |
| #41 | "Electric Stimulation Therapy"[Mesh:NoExp] |
| #42 | gastric*[tiab] OR gastro*[tiab] |
| #43 | #41 AND #42 |
| #44 | #36 OR #37 OR #38 OR #39 OR #40 OR #43 |
| #45 | #17 AND #44 |
| #46 | medline[sb] |
| #47 | Editorial[pt] OR Letter[pt] OR Congress[pt] OR Comment[pt] OR Case Reports[pt] |
| #48 | ("Animals"[Mesh]) NOT "Humans"[Mesh] |
| #49 | #45 NOT #46 |
| #50 | #49 NOT #47 |
| #51 | #50 NOT #48 |

EMBASE:

| #1 | exp abdominal surgery/ or exp gastrointestinal surgery/ |
| --- | --- |
| #2 | exp stomach surgery/ or exp gastrectomy/ or exp gastric bypass surgery/ or exp gastroduodenostomy/ or exp gastroenterostomy/ or exp gastrojejunostomy/ or exp gastropexy/ or exp gastroplasty/ or exp gastrostomy/ or exp gastrotomy/ or exp percutaneous endoscopic gastrostomy/ or exp pyloromyotomy/ or exp pyloroplasty/ or exp pylorus ligation/ or exp stomach fundoplication/ or exp stomach pouch/ |
| #3 | exp roux-en-y gastric bypass/ |
| #4 | exp jejunoileal bypass/ |
| #5 | exp biliopancreatic bypass/ |
| #6 | ((jejunoileal or "jejuno-ilial" or "jejuno ilial" or gastric or anastomosis or anastom* or duoden* or "Roux-Y" or "omega-loop" or omega) adj3 (bypass or bypass*)).ab,kf,kw,ti. |
| #7 | ("roux-en-Y" or LRYGB* or LAGB or gastrectomy or gastroplasty or gastrogastrostomy or "gastro-gastrostomy" or gastroenterostomy).ab,kf,kw,ti. |
| #8 | ((bypass or bypass* or gastrointestinal or malabsorpti* or gastric or "restrictive") adj3 (surg* or operation or operations or intervention or interventions)).ab,kf,kw,ti. |
| #9 | ((gastric or stomach) adj3 (stapl* or plication or imbrication or balloon)).ab,kf,kw,ti. |
| #10 | ((gastrointestinal or biliopancreatic) adj3 diversion*).ab,kf,kw,ti. |
| #11 | ((lap or silicon or gastr*) adj3 (band* or sleeve*)).ab,kf,kw,ti. |
| #12 | ((mason* or malabsorpti*) adj3 procedure*).ab,kf,kw,ti. |
| #13 | (switch* adj3 duoden*).ab,kf,kw,ti. |
| #14 | ((gastro* or gastric or stomach or duoden*) adj3 (surg* or resect* or operation or operations or remov* or incision? or excision?)).ab,kf,kw,ti. |
| #15 | #1 or #2 or #3 or #4 or #5 or #6 or #7 or #8 or #9 or #10 or #11 or #12 or 13 or #14 |
| #16 | exp obesity/ or exp body weight disorder/ or exp abdominal obesity/ or exp diabetic obesity/ or exp morbid obesity/ |
| #17 | exp overnutrition/ or exp nutritional disorder/ |
| #18 | exp body weight/ or exp weight/ |
| #19 | exp body weight loss/ or exp body weight change/ |
| #20 | (obes* or overweight or "over weight" or weightloss or "weight-loss" or "weight los*" or "body-weight" or "bodyweight" or "weight reduction" or "weight red*" or BMI or "body mass index").ab,kf,kw,ti. |
| #21 | exp diabetes mellitus/ or exp "disorders of carbohydrate metabolism"/ or exp diabetic obesity/ or exp insulin dependent diabetes mellitus/ |
| #22 | exp metabolic disorder/ |
| #23 | exp stomach paresis/ or exp stomach function disorder/ or exp diabetic stomach paresis/ |
| #24 | (diabetes* or diabetic* or gastropare* or "metabolic disorder").ab,kf,kw,ti. |
| #25 | #16 or #17 or #18 or #19 or #20 or #21 or #22 or #23 or #24 |
| #26 | #15 and #25 |
| #27 | exp bariatric surgery/ |
| #28 | ((bariatric or obes* or antiobes* or "anti obes*" or "weight reduc*") adj3 (surg* or operation or operations or resect*)).ab,kf,kw,ti. |
| #29 | ("bariatric procedur*" or "bariatric intervention*").ab,kf,kw,ti. |
| #30 | exp gastric pacemaker/ |
| #31 | ((gastric or stomach or gastro*) and (stimulat* or electro* or electrostimulat* or "electro-stimulat*" or electric* or neurostimulat* or neuromudulat* or pacemak* or pace-make* or Medtronic or Transcend* or "Abiliti System*" or "Exilis system*" or "TANTALUS-DIAMOND*" or "TANTALUS*" or MetaCure* or Enterra*)).ab,kf,kw,ti. |
| #32 | #26 or #27 or #28 or #29 or #30 or #31 |
| #33 | exp high volume hospital/ or exp high volume surgeon/ |
| #34 | exp patient volume/ |
| #35 | exp surgical volume/ |
| #36 | exp low volume hospital/ or exp low volume surgeon/ |
| #37 | exp surgeon volume/ |
| #38 | #33 or #34 or #35 or #36 or #37 |
| #39 | (("high-volume*" or "low-volume*" or caseload* or volume or volumes or workload or "case-load" or "work-load" or small or large or type or size) adj3 (hospital or hospitals or clinic or clinics or facility or facilities or center or centers or centre or centres or provider* or institution*)).ab,kf,kw,ti. |
| #40 | ("case volume*" or "service volume*").ab,kf,kw,ti. |
| #41 | (minim* adj2 (volume or volumes or caseload or workload or "case-load" or "work-load")).ab,kf,kw,ti. |
| #42 | ((high-volume* or low-volume* or caseload* or volume or volumes or workload or "case-load" or "work-load") adj3 (surg* or patient* or procedure*)).ab,kf,kw,ti. |
| #43 | ("surgeon experience*" or "surgical experience*" or "surgery experience*" or "surgeon performance" or "surgical performance" or "surgery performance").ab,kf,kw,ti. |
| #44 | exp centralization/ or *decentralization/ or exp organizational restructuring/ or (centrali* or decentrali* or regionali*).ab,kf,kw,ti. |
| #45 | (volume or volumes or caseload* or workload* or "case-load" or "work-load" or hospital* or treat* or service*).ab,kf,kw,ti. |
| #46 | #44 and #45 |
| #47 | exp surgeon/ or (surgeon* or physician* or specialist*).ab,kf,kw,ti. |
| #48 | (caseload* or volume or volumes or workload or "case-load" or "work-load").ab,kf,kw,ti. |
| #49 | #48 and #47 |
| #50 | ((hospital or hospitals or clinic or clinics or facility or facilities or center or centers or centre or centres or provider* or surgeon* or physician* or provider* or specialist* or volume or volumes) adj3 outcome*).ab,kf,kw,ti. |
| #51 | or/#38-#43,#46,#49-#50 |
| #52 | #32 and #51 |
| #53 | exp animal/ not exp human/ |
| #54 | (editorial or erratum or letter or note or patent or conference abstract or Conference Review).pt. |
| #55 | #52 not #53 not #54 |
| #56 | limit #55 to remove medline records |
| #57 | limit #56 to yr="2000 -Current" |

MEDLINE:

| #1 | digestive system surgical procedures/ |
| --- | --- |
| #2 | exp Fundoplication/ |
| #3 | exp anastomosis, roux-en-y/ or biliary tract surgical procedures/ or exp biliopancreatic diversion/ or exp gastrectomy/ or exp gastroenterostomy/ or exp Gastropexy/ or exp Gastroplasty/ or gastrostomy/ or exp pyloromyotomy/ |
| #4 | exp Jejunoileal Bypass/ or exp Gastric Bypass/ |
| #5 | ((jejunoileal or "jejuno-ilial" or "jejuno ilial" or gastric or anastomosis or anastom* or duoden* or "Roux-Y" or "omega-loop" or omega) adj3 (bypass or bypass*)).ab,kf,kw,ti. |
| #6 | ("roux-en-Y" or LRYGB* or LAGB or gastrectomy or gastroplasty or gastrogastrostomy or "gastro-gastrostomy" or gastroenterostomy).ab,kf,kw,ti. |
| #7 | ((bypass or bypass* or gastrointestinal or malabsorpti* or gastric or "restrictive") adj3 (surg* or operation or operations or intervention or interventions)).ab,kf,kw,ti. |
| #8 | ((gastric or stomach) adj3 (stapl* or plication or imbrication or balloon)).ab,kf,kw,ti. |
| #9 | ((gastrointestinal or biliopancreatic) adj3 diversion*).ab,kf,kw,ti. |
| #10 | ((lap or silicon or gastr*) adj3 (band* or sleeve*)).ab,kf,kw,ti. |
| #11 | ((mason* or malabsorpti*) adj3 procedure*).ab,kf,kw,ti. |
| #12 | (switch* adj3 duoden*).ab,kf,kw,ti. |
| #13 | ((gastro* or gastric or stomach or duoden*) adj3 (surg* or resect* or operation or operations or remov* or incision? or excision?)).ab,kf,kw,ti. |
| #14 | #1 or #2 or #3 or #4 or #5 or #6 or #7 or #8 or #9 or #10 or #11 or #12 or #13 |
| #15 | exp Obesity/ or body weight/ or body weight changes/ or weight loss/ or exp overweight/ or exp Obesity, Abdominal/ |
| #16 | diabetes mellitus/ or diabetes mellitus, type 2/ or exp metabolic syndrome/ or Diabetes Mellitus, Type 1/ |
| #17 | exp Overnutrition/ or nutrition disorders/ |
| #18 | (obes* or overweight or "over weight" or weightloss or "weight-loss" or "weight los*" or "body-weight" or "bodyweight" or "weight reduction" or "weight red*" or BMI or "body mass index").ab,kf,kw,ti. |
| #19 | (diabetes* or diabetic* or gastropare* or "metabolic disorder").ab,kf,kw,ti. |
| #20 | #15 or #16 or #17 or #18 or #19 |
| #21 | #14 and #20 |
| #22 | exp bariatric surgery/ |
| #23 | ((bariatric or obes* or antiobes* or "anti obes*" or "weight reduc*") adj3 (surg* or operation or operations or resect*)).ab,kf,kw,ti. |
| #24 | ("bariatric procedur*" or "bariatric intervention*").ab,kf,kw,ti. |
| #25 | ((gastric or stomach or gastro*) and (stimulat* or electro* or electrostimulat* or "electro-stimulat*" or electric* or neurostimulat* or neuromudulat* or pacemak* or pace-make* or Medtronic or Transcend* or "Abiliti System*" or "Exilis system*" or "TANTALUS-DIAMOND*" or "TANTALUS*" or MetaCure* or Enterra*)).ab,kf,kw,ti. |
| #26 | Electric Stimulation Therapy/ |
| #27 | (gastric* or gastro*).ab,kf,kw,ti. |
| #28 | #26 and #27 |
| #29 | #22 or #23 or #24 or #25 or #28 |
| #30 | #21 or #29 |
| #31 | exp Hospitals, High-Volume/ |
| #32 | exp Hospitals, Low-Volume/ |
| #33 | (("high-volume*" or "low-volume*" or caseload* or volume or volumes or workload or "case-load" or "work-load" or small or large or type or size) adj3 (hospital or hospitals or clinic or clinics or facility or facilities or center or centers or centre or centres or provider* or institution*)).ab,kf,kw,ti. |
| #34 | ("case volume*" or "service volume*").ab,kf,kw,ti. |
| #35 | (minim* adj2 (volume or volumes or caseload or workload or "case-load" or "work-load")).ab,kf,kw,ti. |
| #36 | ((high-volume* or low-volume* or caseload* or volume or volumes or workload or "case-load" or "work-load") adj3 (surg* or patient* or procedure*)).ab,kf,kw,ti. |
| #37 | ("surgeon experience*" or "surgical experience*" or "surgery experience*" or "surgeon performance" or "surgical performance" or "surgery performance").ab,kf,kw,ti. |
| #38 | (centrali* or decentrali* or regionali*).ab,kf,kw,ti. |
| #39 | (volume or volumes or caseload* or workload* or "case-load" or "work-load" or hospital* or treat* or service*).ab,kf,kw,ti. |
| #40 | #38 and #39 |
| #41 | exp Surgeons/ or (surgeon* or physician* or specialist*).ab,kf,kw,ti. |
| #42 | (caseload* or volume or volumes or workload or "case-load" or "work-load").ab,kf,kw,ti. |
| #43 | #41 and #42 |
| #44 | ((hospital or hospitals or clinic or clinics or facility or facilities or center or centers or centre or centres or provider* or surgeon* or physician* or provider* or specialist* or volume or volumes) adj3 outcome*).ab,kf,kw,ti. |
| #45 | #31 or #32 or #33 or #34 or #35 or #36 or #37 or #40 or #43 or #44 |
| #46 | #30 and #45 |
| #47 | exp Animals/ not exp Humans/ |
| #48 | comment/ or editorial/ or consensus/ or exp guideline/ |
| #49 | (case reports or comment or congress or editorial or guideline or letter).pt. |
| #50 | case report.mp. |
| #51 | #46 not #47 not #48 not #49 not #50 |
| #52 | limit #51 to yr="2000 -Current" |

**Appendix 2: Excluded full-texts**

Context: (29)

| Akpinar, E. O., et al. (2022). "Hospital Variation in Preference for a Specific Bariatric Procedure and the Association with Weight Loss Performance: a Nationwide Analysis." Obesity Surgery 32(11): 3589-3599. |
| --- |
| Amato, L., et al. (2013). "Volume and health outcomes: evidence from systematic reviews and from evaluation of Italian hospital data." Volumi di attivita ed esiti delle cure: prove scientifiche dalla letteratura e dalle valutazioni empiriche in Italia. 37(2-3 Suppl 2): 1-100. |
| Amato, L., et al. (2017). "Volume and health outcomes: evidence from systematic reviews and from evaluation of Italian hospital data." Volumi di attivita ed esiti delle cure: prove scientifiche in letteratura ed evidenze empiriche in Italia. 41(5-6 (Suppl 2)): 1-128. |
| Consortia Editorial Office (2000). "Guideliness for laparoscopic and open surgical treatment of morbid obesity." Obesity Surgery 10(4): 378-379. |
| Daskalakis, M., et al. (2011). "Impact of surgeon experience and buttress material on postoperative complications after laparoscopic sleeve gastrectomy." Surgical Endoscopy 25(1): 88-97. |
| Dayer-Jankechova, A., et al. (2016). "Complications After Laparoscopic Roux-en-Y Gastric Bypass in 1573 Consecutive Patients: Are There Predictors?" Obesity Surgery 26(1): 12-20. |
| Dimick, J. B., et al. (2014). "Composite measures for profiling hospitals on bariatric surgery performance." JAMA surgery 149(1): 10-16. |
| Domenghino, A., et al. (2023). "Delivering Safe Surgical Care While Simultaneously Caring for Patients With COVID-19; Assessment of Patient Selection, Volume and Outcomes in a Tertiary Care Hospital." International journal of public health 68: 1605640. |
| Fischer, L., et al. (2022). "Developing a competence center for obesity and metabolic surgery-Experiences from two different clinics." Der Weg zum Kompetenzzentrum fur Adipositas und metabolische Chirurgie - Erfahrungen aus 2 verschiedenen Kliniken. 93(9): 876-883. |
| Harrison, E. M., et al. (2016). "Individual surgeon mortality rates: can outliers be detected? A national utility analysis." BMJ Open 6(10): e012471. |
| Lee, A. N., et al. (2018). "Outcomes at bariatric surgery centers of excellence and non-designated centers: A retrospective cohort study in a TRICARE population." American Surgeon 84(3): 410-415. |
| Leeman, M., et al. (2020). "The Influence of Surgical Experience on Postoperative Recovery in Fast-Track Bariatric Surgery." Obesity Surgery 30(5): 1653-1659. |
| Lindsey, M. L., et al. (2009). "Bariatric surgery for obesity: surgical approach and variation in in-hospital complications in New York State." Obesity Surgery 19(6): 688-700. |
| Liu, J. B., et al. (2017). "Concurrent bariatric operations and association with perioperative outcomes: Registry based cohort study." BMJ (Online) 358: 4244. |
| Livingston, E. H. (2010). "The incidence of bariatric surgery has plateaued in the U.S." American Journal of Surgery 200(3): 378-385. |
| McGlone, E. R., et al. (2023). "Bariatric surgery provision in response to the COVID-19 pandemic: retrospective cohort study of a national registry." Surgery for obesity and related diseases : official journal of the American Society for Bariatric Surgery. |
| Nguyen, N. T., et al. (2005). "Improving the quality of care in bariatric surgery: the volume and outcome relationship." Advances in surgery 39: 181-191. |
| Nguyen, N. T., et al. (2012). "Outcomes of bariatric surgery performed at accredited vs nonaccredited centers." Journal of the American College of Surgeons 215(4): 467-474. |
| Pratt, G. M., et al. (2009). "Demographics and outcomes at American Society for Metabolic and Bariatric Surgery Centers of Excellence." Surgical Endoscopy 23(4): 795-799. |
| Shabbir, A. and D. Dargan (2015). "The success of sleeve gastrectomy in the management of metabolic syndrome and obesity." Journal of Biomedical Research 29(2): 93-97. |
| Shah, R. M., et al. (2023). "Effects of Geographic Region, Hospital Volume, and Teaching Status on Perioperative Outcomes in Bariatric Surgery." Journal of gastrointestinal surgery : official journal of the Society for Surgery of the Alimentary Tract. |
| Stepaniak, P. S., et al. (2012). "Bariatric surgery with operating room teams that stayed fixed during the day: a multicenter study analyzing the effects on patient outcomes, teamwork and safety climate, and procedure duration." Anesthesia and analgesia 115(6): 1384-1392. |
| Stroh, C., et al. (2017). "Does Certification as Bariatric Surgery Center and Volume Influence the Outcome in RYGB-Data Analysis of German Bariatric Surgery Registry." Obesity Surgery 27(2): 445-453. |
| Svarts, A., et al. (2020). "Does Focus Improve Performance in Elective Surgery? A Study of Obesity Surgery in Sweden." International journal of environmental research and public health 17(18). |
| Varban, O. A., et al. (2021). "Evaluating the Effect of Surgical Skill on Outcomes for Laparoscopic Sleeve Gastrectomy: A Video-based Study." Annals of Surgery 273(4): 766-771. |
| Varban, O. A., et al. (2020). "Surgeon variation in severity of reflux symptoms after sleeve gastrectomy." Surgical Endoscopy 34(4): 1769-1775. |
| Wilkinson, K. H., et al. (2021). "The Effect of Bariatric Surgery Volume on General Surgery Outcomes for Morbidly Obese Patients." Journal of obesity 2021: 8945091. |
| Zambare, W. V., et al. (2021). "Outcomes in Laparoscopic Roux-en-Y Gastric Bypass and Implications for Surgical Resident Education." Journal of surgical education 78(6): e161-e168. |
| Zhang, L., et al. (2017). "Changes in utilization and peri-operative outcomes of bariatric surgery in large U.S. hospital database, 2011-2014." PLoS ONE 12(10): e0186306. |

Population: (9)

| Altieri, M. S., et al. (2016). "Robotic-assisted outcomes are not tied to surgeon volume and experience." Surgical Endoscopy 30(7): 2825-2833. |
| --- |
| Blackburn, K. W., et al. (2023). "Monitoring performance in laparoscopic gastric bypass surgery using risk-adjusted cumulative sum at 2 high-volume centers." Surgery for obesity and related diseases : official journal of the American Society for Bariatric Surgery. |
| Brunaud, L., et al. (2018). "Health Care Institutions Volume Is Significantly Associated with Postoperative Outcomes in Bariatric Surgery." Obesity Surgery 28(4): 923-931. |
| Courcoulas, A., et al. (2003). "The relationship of surgeon and hospital volume to outcome after gastric bypass surgery in Pennsylvania: a 3-year summary." Surgery 134(4): 613-613. |
| Kyler, K. E., et al. (2019). "Trends in Volume and Utilization Outcomes in Adolescent Metabolic and Bariatric Surgery at Children's Hospitals." The Journal of adolescent health : official publication of the Society for Adolescent Medicine 65(3): 331-336. |
| Mlaver, E. and J. Sharma (2023). "Which Procedures Contribute Most to the System-Wide Burden of Postoperative Venous Thromboembolism?" American Surgeon. |
| Mustafa, M., et al. (2023). "THE IMPACT OF SURGEON VOLUME ON PATIENT OUTCOMES IN COMPLEX SURGICAL PROCEDURES." NeuroQuantology 21(6): 55-60. |
| Nimptsch, U., et al. (2019). "Complex gastric surgery in Germany-is centralization beneficial? Observational study using national hospital discharge data." Langenbeck's Archives of Surgery 404(1): 93-101. |
| Reavis, K. M., et al. (2009). "Hospital volume is not a predictor of outcomes after gastrectomy for neoplasm." The American surgeon 75(10): 932-936. |

Intervention: (1)

| Biancari, F., et al. (2012). "Individual surgeon's impact on the risk of re  exploration for excessive bleeding after coronary artery bypass surgery." Journal of cardiothoracic and vascular anesthesia 26(4): 550-556. |
| --- |

**Appendix 3: Volume definitions stratified by volume type**

| **Surgeon volume** | | | | | | | | |
| --- | --- | --- | --- | --- | --- | --- | --- | --- |
| **Author, year of publication** | **Sample size** | **Case volume categories** | | | **Case volume** | **Total no. of patients/Case volume category** | | **Total no. of units/Case volume category** |
| Altieri 2020 [20] | 46 511 | Multiple changepoints | | | NR | NR | | NR |
| Celio 2016 [39] | 16 547 | Volume of study year | | Low | <50 | SG: 9 083; RYGB: 6 588 | | SG: 649; RYGB: 555 |
|  |  |  |  | High | ≥ 50 | SG: 7 464; RYGB: 2 495 | | SG: 87; RYGB: 181 |
| Celio 2017 [18] | 32 521 | - | | Low | <50 | 11 142 | | 617 |
|  |  |  |  | High | ≥50 | 21 379 | | 216 |
| Chao 2021 [40] | 27 714 | Percentiles / Annual volume | | 5^th^ | Averages: 10 | Averages: 10 | | NR |
|  |  |  |  | 10^th^ | 16 | 16 | |  |
|  |  |  |  | 25^th^ | 38 | 38 | |  |
|  |  |  |  | 50^th^ | 77 | 77 | |  |
|  |  |  |  | 75^th^ | 133 | 133 | |  |
| Hunt 2020 [41] | 13 836 | Unnamed categories / Annual volume | | <75 | <75 | 2 366 | | NR |
|  |  |  |  | 125-75 | 125-75 | 6 890 | |  |
|  |  |  |  | >125 | >125 | 4 405 | |  |
| Lopez 2002 [42] | 933 | Monthly operational frequency | | <2/month | <2/month | No. of operations: 169 | | 34 |
|  |  |  |  | ≥2/month | ≥2/month | 764 | | 10 |
| Smith 2010 [37] | 3410 | Unnamed categories / Midpoint of lifetime volume | | <25 | <25 | 352 | | 9 |
|  |  |  |  | 25-50 | 25-50 | 461 | | 6 |
|  |  |  |  | 50-100 | 50-100 | 1 219 | | 9 |
|  |  |  |  | ≥100 | ≥100 | 1 378 | | 7 |
| Smith 2013 [38] | 3 410 | Unnamed categories / Continuous volume (1 unit=10 cases/year) | | <25 | <25 | 352 | | 9 |
|  |  |  |  | 25-50 | 25-50 | 461 | | 6 |
|  |  |  |  | 50-100 | 50-100 | 1 219 | | 9 |
|  |  |  |  | ≥100 | ≥100 | 1 378 | | 7 |
| **Hospital and surgeon volume** | | | | | | | | |
| **Author, year of publication** | **Sample size** | **Case volume categories** | | | **Case volume** | **Total no. of patients/ Case volume category** | | **Total no. of units/ Case volume category** |
| Bouchard 2020 [59] | 2 623 | Incremental analysis | | Incremental analysis | 1 unit = 10 cases | Surgeon median: 33 in 2012;  High volume hospitals: 154/year | | NR |
| Chadwick 2023 [19] | 63 604 | Categories / Continuous volume (decrease in volume) | | Low | Hospitals: <50;  Surgeons: <20 | Hospitals: 5 108;  Surgeons: 3 837 | | NR |
|  |  |  |  | Intermediate | Hospitals: 120-50; Surgeons: 50-20 | Hospitals: 15 779;  Surgeons: 8 469 | |  |
|  |  |  |  | High | Hospitals: >120;  Surgeons: >50 | Hospitals: 42 694;  Surgeons: 51 275 | |  |
| Chiu 2012 [60] | 2 674 | Annual volume | | Low | Hospitals: <35;  Surgeons: <15 | Hospitals: 1 770;  Surgeons: 1 767 | | Hospitals: 262; Surgeons: 392 |
|  |  |  |  | High | Hospitals: ≥35;  Surgeons: ≥15 | Hospitals: 904; Surgeons: 907 | | Hospitals: 14; Surgeons: 43 |
| Doumouras 2017 [61] | 13 256 | Hospitals: Unnamed categories / Surgeons: 1 unit = 25 | | Hospitals: <200; | Hospitals: <200 | Hospitals: 2 364;  Surgeon mean annual: 100 IQR (86-138) | | NR |
|  |  |  |  | 400-200 | Hospitals: 400-200 | Hospitals: 4 698 | |  |
|  |  |  |  | >400 | Hospitals: >400 | Hospitals: 6 194 | |  |
| Hollenbeak 2008 [12] | 14 716 | Annual volume | | Low | Hospitals & Surgeons: <50 | Hospitals: 2 158;  Surgeons: NR | | Hospitals: 26-50 (1999-2003) |
|  |  |  |  | Medium | Hospitals & Surgeons: 50-100 | Hospitals: 1 835;  Surgeons: NR | | Hospitals: 35-54 (1999-2003) |
|  |  |  |  | High | Hospitals & Surgeons: >100 | Hospitals: 10 723;  Surgeons: NA | | Hospitals: 43-64 (1999-2003) |
| Murr 2007 [62] | 19 174 | 5-year procedure volumes groups | | Hospitals: 5 volume groups; Surgeons: 4 Volume groups | Hospitals: 1-9; Surgeons: 6-99 | Hospitals: NR;  Surgeons: 1832 | | Hospitals: 29; Surgeons: 57 |
|  |  |  |  | Hospitals: Group 2; Surgeons: Group 2 | Hospitals: 10-99; Surgeons: 100-199 | Hospitals: 1 340;  Surgeons: 1 392 | | Hospitals: 31; Surgeons: 10 |
|  |  |  |  | Hospitals: Group 3; Surgeons: (No Group) | Hospitals: 100-199; | Hospitals: 1 321 | | Hospitals: 10 |
|  |  |  |  | Hospitals: Group 4; Surgeons: Group 3 | Hospitals: 200-499; Surgeons: 200-499 | Hospitals: 3 198;  Surgeons: 6 407 | | Hospitals: 9; Surgeons: 17 |
|  |  |  |  | Hospitals: Group 5; Surgeons: Group 4 | Hospitals: ≥500 Surgeons: ≥500 | Hospitals: 13 213;  Surgeons: 9 390 | | Hospitals: 14; Surgeons: 12 |
| Torrente 2013 [13] | 14 714 | Annual volume | | Low | Hospitals: <125; Surgeons: <50 | Hospitals: 4 871;  Surgeons: 3 670 | | NR |
|  |  |  |  | Medium | Hospitals: 125-299 | Hospitals: 5 491 | |  |
|  |  |  |  | High | Hospitals: ≥300; Surgeons: ≥50 | Hospitals: 4 649;  Surgeons: 11 044 | |  |
| Kelles 2009 [63] | 74 774 | Annual volume / Unnamed categories | | Hospitals: <20  Surgeons: <20 | Hospitals: <20; Surgeons: <20 | Hospitals: 669;  Surgeons: 368 | | Hospitals: 11; Surgeons: 41 |
|  |  |  |  | Hospitals: ≥20;  Surgeons: ≥20 | Hospitals: ≥20; Surgeons: ≥20 | Hospitals: 1 498;  Surgeons: 1 799 | | Hospitals: 5; Surgeons: 7 |
| **Hospital volume** | | | | | | | | |
| **Author, year of publication** | **Sample size** | **Case volume categories** | | | **Case volume** | | **Total no. Of patients/Case volume category** | **Total no. of units/ Case volume category** |
| Carbonell 2005 [43] | 5 786 | Annual volume | Very low | | <50 | | Discharges: 1 212 | 105 |
|  |  |  | Low | | 50-99 | | 838 | 12 |
|  |  |  | Medium | | 100-199 | | 1 845 | 14 |
|  |  |  | High | | >200 | | 2 072 | 6 |
| Dimick 2009 [44] | 16 221 | Quartiles / Volume of 2 years | 1st | | <29 | | 4 090 | 44 |
|  |  |  | 2^nd^ | | 29-98 | | 4 188 | 23 |
|  |  |  | 3^rd^ | | 99-209 | | 4 102 | 8 |
|  |  |  | 4^th^ | | >209 | | 3 841 | 5 |
| Encinosa 2009 [45] | 2001-2002: 2 522; 2005-2006: 7 060 | Terciles | Low | | <159 | | 2001-2002: 44.45%; 2005-2006: 32.11% | NR |
|  |  |  | Medium | | 521-160 | | 2001-2002: 26.36%; 2005-2006: 35.57% |  |
|  |  |  | High | | >521 | | 2001-2002: 29.18%; 2005-2006: 32.32% |  |
| Gould 2011 [46] | 2 509 | Progressive groups compared with lower groups / Annual volume of every year | ≥25 | | ≥25 | | NR | NR |
|  |  |  | ≥50 | | ≥50 | |  |  |
|  |  |  | ≥75 | | ≥75 | |  |  |
|  |  |  | ≥100 | | ≥100 | |  |  |
|  |  |  | ≥125 | | ≥125 | |  | 2005: 27; 2006: 33; 2007: 26 |
|  |  |  | ≥150 | | ≥150 | |  | NR |
|  |  |  | ≥175 | | ≥175 | |  |  |
|  |  |  | ≥200 | | ≥200 | |  |  |
| Hernandez-Boussard 2012 [15] | 354 478 | Terciles / Annual volume | Low | | <89 | | 101 548 | NR |
|  |  |  | Mid | | 89-222 | | 174 830 |  |
|  |  |  | High | | >222 | | 78 100 |  |
| Ibrahim 2017 [47] | 145 527 | Terciles / Annual volume | Low | | NA | | Annual mean (sd): 156 (20) | 55 |
|  |  |  | Medium | | NA | | 239 (27) | 55 |
|  |  |  | High | | NA | | 448 (131) | 55 |
| Jafari 2013 [48] | 277 760 | Annual volume | Low | | <50 | | 41 547 | 484 ± 50 |
|  |  |  | High | | ≥50 | | 236 219 | 328 ± 48 |
| Kauppila 2020 [14] | 49 977 | Terciles / Annual volume | Lowest | | <7 | | 1692 | NR |
|  |  |  | Medium | | 25-7 | | 6590 |  |
|  |  |  | Highest | | ≥26 | | 32 919 |  |
| Kohn 2010 [49] | 102 069 | Annual volume / Continuous volume | <100 | | <100 | | NR | 90-143 (1998-2006) |
|  |  |  | 125-100 | | 125-100 | | NR | 2-13 (1998-2006) |
|  |  |  | >125 | | >125 | | NR | 1-39 (1998-2006) |
| Krell 2014 [50] | 31 240 | Prior caseload thresholds / Annual volume | Lowest | | 50 | | 3 781 | 67 |
|  |  |  | Medium | | 100 | | 7 780 | 65 |
|  |  |  | Highest | | 125 | | 19 679 | 66 |
| Liu 2003 [51] | 16 232 | Annual volume | Very low | | <50 | | 2 314 | 81 |
|  |  |  | Low | | 50-99 | | 3 067 | 9 |
|  |  |  | Medium | | 100-199 | | 4 240 | 7 |
|  |  |  | High | | ≥200 | | 6 611 | 4 |
| Markar 2023 [52] | 77 870 (68 084 Sweden; 9 786 Finland) | Quartiles and continuous volume / Annual volume | Quartile 1 | | <55.5 | | 19 471 | NR |
|  |  |  | Quartile 2 | | 55.75-111.25 | | 19 548 |  |
|  |  |  | Quartile 3 | | 111.5-221.5 | | 19 514 |  |
|  |  |  | Quartile 4 | | >221.5 | | 19 337 |  |
| Morino 2007 [53] | 13 431 | - | Low | | <100 | | NR | NR |
|  |  |  | High | | >100 | |  |  |
| Nguyen 2004 [54] | 24 166 | Annual volume | Low | | <50 | | 2 722 | 44 |
|  |  |  | Medium | | 100-50 | | 7 634 | 27 |
|  |  |  | High | | >100 | | 13 810 | 22 |
| Pradarelli 2016 [55] | 8 693 | Annual volume | Low | | <50 | | 214 | 7 |
|  |  |  | Medium | | 124-50 | | 2 083 | 17 |
|  |  |  | High | | ≥125 | | 6 396 | 16 |
| Stenberg 2014 [56] | 25 038 | Annual volume | <100 | | <100 | | 2 835 | NR |
|  |  |  | 100-149 | | 100-149 | | 1 526 |  |
|  |  |  | 150-199 | | 150-199 | | 2 108 |  |
|  |  |  | 299-249 | | 299-249 | | 2 139 |  |
|  |  |  | 259-299 | | 259-299 | | 1 792 |  |
|  |  |  | >300 | | >300 | | 8 337 |  |
| Svarts 2022 [16] | 52 703 | 1 unit = 100 cases | | | 1 unit = 100 cases | | Average volume: 30-116 | 51 hospitals overall |
| Tsui 2020 [17] | 8 389 | Annual volume | Low | | <45 | | 2969 | NR |
|  |  |  | Medium | | 45-65 | | 2537 |  |
|  |  |  | High | | >65 | | 2883 |  |
| Varban 2015 [57] | 446 127 | Unnamed categories / Annual volume | <50 | | <50 | | 2006-2007: 41 139;  2008-2009: 44 064;  2010-2011: 50 026 | NR |
|  |  |  | 50-125 | | 50-125 | | 2006-2007: 9 623;  2008-2009: 20 465;  2010-2011: 15 057 |  |
|  |  |  | >125 | | >125 | | 2006-2007: 9 884;  2008-2009: 20 465;  2010-2011: 7 736 |  |
| Wilson 2015 [58] | 256 694 overall, GB: 62 010 | Terciles / Annual volume | Low | | NR | | NR | NR |
|  |  |  | Medium | |  |  |  |  |
|  |  |  | High | |  |  |  |  |

Abbreviations: NR - Not reported; SG - sleeve gastrectomy; RYGB - Roux-en Y gastric bypass; sd - standard deviation

**Appendix 4: Summary of adjusted study results, incl. procedure type, high-volume cutoffs and quality appraisal**

| **First author, year of publication** | **Procedure ^†^** | **Mortality outcomes** | | | **Other outcomes** | | | **High-Volume Category ^⸸^** | **Quality points** |
| --- | --- | --- | --- | --- | --- | --- | --- | --- | --- |
|  |  | Hospital Mortality | Short-term Mortality | Long-term/intermediate Mortality | Morbidity | Disease-related morbidity | LOS |  |  |
| Chadwick 2023 [19] | SG |  |  |  |  |  | +/++ | Incremental | 16.5 |
|  | RYGB |  |  |  |  |  | +/++ |  |  |
|  | OAGB |  |  |  |  |  | ++ |  |  |
|  | LAGB |  |  |  |  |  | +/++ |  |  |
| Altieri 2020 [20] | RYGB |  |  |  | ++ ^A1^ |  | ++ ^B1^ | Changepoints | 16.5 |
|  | LSG |  |  |  | ++ ^A2^ |  | ++ ^B2^ |  |  |
| Svarts 2022 [16] | GB, GS |  |  |  | + ^C^ | + | + | 100 case increments | 15 |
| Bouchard 2020 [59] | SG |  | +/**○○** ^1^ |  |  | & |  | 10 case increments | 14 |
|  | RYGB |  | **○**/**○○** ^1^ |  |  | & |  |  |  |
| Torrente 2013 [13] | Gastroenterostomy without gastrectomy, high GB, other gastroenterostomy | +/++ | +/++ |  |  |  |  | H: ≥300;  S: ≥50 | 14 |
| Kauppila 2020 [14] | GB, VBG, GBa, other bariatric procedure, malabsorptive procedure | + ^2^ |  |  | & |  |  | H: ≥26 | 14 |
| Markar 2023 [52] | Bariatric surgery |  |  | + ^D^ |  |  |  | H: >221.5 | 14 |
| Hollenbeak 2008 [12] | Gastroenterostomy without gastrectomy, high GB; other gastroenterostomy | +/++ | +/++ |  |  |  | +/++ | H: >100;  S: >100 | 13.5 |
| Pradarelli 2016 [55] | LSG |  |  |  | **○** |  |  | H: ≥125 | 13.5 |
| Wilson 2015 [58] | GB |  |  |  | + |  |  | NR | 13.5 |
| Encinosa 2009 [45] | GBa or gastroplasty without GB, RYGB, other types of GB, laparoscopic banding or gastroplasty without GB, banding and gastroplasty without bypass; GB |  |  |  | + ^E^ |  |  | H: >521 | 13 |
| Stenberg 2014 [56] | LGB |  |  |  | + |  |  | H: >300 | 12.5 |
| Kohn 2010 [49] | GB, LAGB, gastroplasty, malabsorptive procedures, LAGB | + |  |  |  |  |  | H: >125 | 12.5 |
| Jafari 2013 [48] | LRYGB, LSG | + |  |  | + |  |  | H: ≥50 | 12 |
| Gould 2011 [46] | GB, LGB, LAGB | + ^3^ |  |  | & |  |  | H: ≥200 | 12 |
| Chao 2021 [40] | LRYGB |  |  |  | ⨁⨁ ^F^ |  |  | S: >133 | 11.5 |
| Varban 2015 [57] | LAGB | **○** ^4^ |  |  | ⨁ ^G^ |  |  | H: >125 | 10 |
|  | LRYGB | **○** ^4^ |  |  | ⨁ ^G^ |  |  |  |  |
| Carbonell 2005 [43] | GB |  |  |  |  | **○** |  | H: >200 | 10 |
| Doumouras 2017 [61] | GB, SG |  |  |  |  | +/**○○** |  | H: >400; S: 20 case increments | 9.5 |
| Murr 2007 [62] | High GB, Gastroenterostomy |  |  |  | +/++ |  |  | H: ≥500;  S: ≥500 | 9.5 |
| Liu 2003 [51] | GB |  |  |  | + |  |  | H: ≥200 | 9.5 |
| Hunt 2020 [41] | RYGB, SG, BPD |  |  |  |  | ++ |  | S: >125 | 8.5 |
| Chiu 2012 [60] | GB, incl. MGB, RYGB, LGB (including laparoscopic MGB), LRYGB, open gastroplasty (including VBG), laparoscopic gastroplasty (incl. LVBG), LAGB |  |  |  |  |  | +/++ | H: ≥35;  S: ≥15 | 8.5 |
| Dimick 2009 [44] | GB, gastroplasty, LGB |  |  |  |  | + |  | H: >209 | 8.5 |
| Smith 2010 [37] | RYGB |  | ++^5^ |  |  |  |  | S: ≥100 | 7.5 |
| Celio 2016 [39] | RYGB, SG |  |  |  | ⨁⨁ ^H^ |  |  | S: ≥ 50 | 7.5 |
| Celio 2017 [18] | LRYGB |  |  |  | ++ |  |  | S: ≥ 50 | 7.5 |
| Smith 2013 [38] | RYGB |  | ++ ^I,5^ |  |  |  |  | S: 10 case increments | 6.5 |
| Hernandez-Boussard 2012 [15] | RYGB | + ^J^ |  |  | ⨁ ^K^ |  |  | H: >222 | 6.5 |
| Kelles 2009^×^ [63] | RYGB |  | ++ |  |  |  | ++ | H: ≥20;  S: ≥20 | 6.5 |
| **significant:** favoring high-volume hospital **+** / surgeon **++** ; **not significant:** hospital **○** / surgeon **○○**; **Mixed effect:** hospital: ⨁ / surgeon: ⨁⨁; **Same result from composite outcome including mortality**: &; NA: Not applicable; NR: Not reported; Note: Studies appraised above the median quality score are denoted by a **bolt** line ^†^ More Details on procedures in Table 1; ^⸸^ Cutoff for the defined high-volume for H: Hospital volume; S: Surgeon volume; ^×^ Intended to analyze hospital volume but excluded in modelling; ^1^: Mortality included with composite outcome; ^2^: Composite hospital mortality and reintervention; ^3^: Occurrence of one or more sever post-operative in-hospital complications and in-hospital mortality; ^4^: Results consistent for 3 different time periods separately analyzed ^5^: Composite event including death; ^A1^: Readmission: improvement up to a volume of 354/Complications: improvement up to a volume of 248 ^A2^: Readmission: improvement up to a volume of 138/ Complications: improvement up to a volume of 62; ^B1^: Improvement until volume of 62; ^B2^: Improvement until volume of 26; ^C^: Intra-operative, 30 day and one year complications; ^D^: Not significant in continuous analysis; ^E^: Postoperative complications and hospital visits with complications; ^F^: Significant for any complication, not significant for single complications; ^G^: Mixed results for different time-cohorts; ^H^: Mixed results for complications, readmissions and reoperations; ^I^: Including FTR in separate analysis; ^J^: Continuous Analysis; ^K^: Significant for multiple complications, not significant for postoperative sepsis | | | | | | | | | |

Abbreviations: GB – Gastric bypass; GBa – Gastric Banding; GS – Gastric sleeve; LAGB - laparoscopic adjustable gastric band; LRYGB - laparoscopic Roux-en-Y gastric bypass; LSG – laparoscopic sleeve gastrectomy; LVGB – VBG – Vertical banded gastroplasty; MGB – Mini gastric bypass; OAGB - one-anastomosis gastric bypass; RYGB – Roux-en-Y gastric bypass; SG – Sleeve gastrectomy; VBG – Vertical banded gastroplasty;

**Appendix 5: Other outcomes stratified by volume types**

| **Surgeon volume** | | | | | |
| --- | --- | --- | --- | --- | --- |
| **Author, year of publication** | **Morbidity**  *Perioperative morbidity/ adverse events of the intervention/ complications*: including re-intervention and re-admission | **Morbidity**  (long term-) Weight reduction; (long term-) BMI reduction; Remission rate of comorbidities (diabetes mellitus, arterial hypertension, sleep apnoea, hyperlipidaemia, etc.) | **Length of stay (LOS)**  (Hospitals; ICU) | **Quality appraisal** | |
|  |  |  |  | **ISPOR** (Yes/Partially/No/NA) | **ROBINS-E**  (Domain 7†) |
| Altieri 2020 [20] | **LRYGB: 30-day readmissions:** (aOR): improvement of outcomes until volume of 354: (0.99 0.98–0.99) \| L**RYGB: peri-operative complications:** (aOR): improvement of outcomes until volume of 248: 0.97 (0.96-0.98) \| **LSG: 30-day readmissions:** (aOR): risk increased after volume of 138: 1.1 (1.0–1.21) \| **LSG: peri-operative complications:** (aOR): improvement of outcomes until volume of 62: 0.94 (0.91-0.98) |  | **Hospitals:** LRYGB: prolonged LOS: (aOR): improvement until volume of 62:  0.9 (0.85-0.95) \| **Hospitals:** LSG: prolonged LOS: (aOR): improvement until volume of 26:  0.82 (0.72-0.93)\| | (10/5/5/7) | Low |
| Bouchard 2020* [59] | **SG: 90-day major morbidity** (aOR, 1 unit = 10 cases): 0.99 (0.93–1.06);  **RYGB: 90-day major morbidity** (aOR, 1 unit = 10 cases): 0.82 (0.71–0.94)  {Included: complications: bleeding, venous thromboembolic event, pneumonia, macrovascular events (myocardial infarction and strokes), postoperative infection/leak, shock, need for de novo hemodialysis, reintubation, prolonged intubation (>48 hours), prolonged length of stay (LOS; ≥7 days), or ***mortality***} |  |  | (10/8/2/7) | Very High |
| Celio 2016 [39] | **Low volume SG-surgeons: Complications:** (30 day, aOR): Low (RYGB) vs. High: 0.955 (0.97-1.14) \| **Readmission:** (30 day, aOR): Low (RYGB) vs. High: 1.303 (1.03-1.64) \| **Reoperation:** (30 day, aOR): Low (RYGB) vs. High: 1.1 (0.76-1.57)\| **High volume SG-surgeons: Complications:** (30 day, aOR): Low (RYGB) vs. High: 0.61 (0.48-0.76) \| **Readmission:** (30 day, aOR): Low (RYGB) vs. High: 0.76 (0.55-1.03) \| **Reoperation:** (30 day, aOR): Low (RYGB) vs. High: 0.45 (0.25-0.78) |  |  | (7/1/12/7) | High |
| Celio 2017 [39] | **Readmission:** (30 day, aOR): Low vs. High: 0.85 (0.77-0.94) \| **Reoperation:** (30 day, aOR): Low vs. High: 0.82 (0.72-0.93) \| **Complication:** (30 day, aOR): Low vs. High: 0.81 (0.75-0.87) \| **Anastomotic leak:** (30 day, aOR): Low vs. High: 0.64 (0.46-0.87) |  |  | (5/2/13/7) | High |
| Chadwick 2023*[19] |  |  | **Hospitals:** Average LOS: (adjusted beta regression weights, incremental DECREASE of volume):  SG: 0.182 days (0.153–0.21) days \|  RYGB: 0.312 days (0.22-0.405) days \| OAGB: 0.22 days (0.047–0.393) days \|  LAGB: 0.141 days (0.093–0.188) days | (12/5/3/7) | Low |
| Chao 2021 [40] | **Any complication**: (30 day, adjusted percentage): 5th (volume percentile, lowest): 6.71%; 10th: 6.65%; 25th: 6.42%; 50th: 6.05%; 75th: 5.55%, p=0.01 \| **Reoperation due to hemorrhage:** (30 day, adjusted percentage): 5th (volume percentile, lowest): 0.13%; 10th: 0.12%; 25th: 0.09%; 50th: 0.06%; 75th: 0.03%, p=0.203 \| **Reoperation due to leak/organ injury:** (30 day, adjusted percentage): 5th (volume percentile, lowest): 0.13%; 10th: 0.13%; 25th: 0.12%; 50th: 0.1%; 75th: 0.07%, p=0.262 \| **Reoperation due to deep infection:** (30 day, adjusted percentage): 5th (volume percentile, lowest): 0.46%; 10th: 0.46%; 25th: 0.43%; 50th: 0.39%; 75th: 0.33%, p=0.110 \| **Readmission:** (30 day, adjusted percentage): 5th (volume percentile, lowest): 3.52%; 10th: 3.51%; 25th: 3.51%; 50th: 3.5%; 75th: 3.49%, p=0.923 \| **Readmission:** (1 year day, adjusted percentage): 5th (volume percentile, lowest): 13.9%; 10th: 13.85%; 25th: 13.67%; 50th: 13.35%; 75th: 12.9%, p=0.099 \| **Revision:** (1 year day, adjusted percentage): 5th (volume percentile, lowest): 0.65%; 10th: 0.64%; 25th: 0.61%; 50th: 0.55%; 75th: 0.48%, p=0.193; |  |  | (10/3/7/7) | High |
| Chiu 2012* [60] |  |  | **Hospitals:** (days, adjusted beta hierarchical regression weights): Low vs. High: -4.23 (-0.72), p<0.001 | (8/1/11/7) | High |
| Doumouras 2017* [61] |  | **All cause morbidity:** (aOR): (per 25 cases): 0.94 (0.88–1.00) |  | (7/5/8/7) | High |
| Hollenbeak 2008* [12] |  |  | **Hospitals:** (undefined): (GLM; identity link: Beta weight): High vs. Medium: 0.26, p<0.0001; Low: 1.24, p<0.0001 | (9/5/6/7) | Low |
| Hunt 2020 [41] |  | **Morbidity:** (aOR): <75 vs. 75-125: 0.82 (0.69–0.98); >125: 0.71 (0.54–0.91) |  | (6/5/9/7) | Low |
| Kelles 2009* [63] |  |  | **Hospitals:** (95%-ile, 5 days): (aOR): High vs. Low: 3.76 (2.54–5.56) | (5/3/12/7) | High |
| Lopez 2002 [42] |  |  | **Hospitals:** (days): <2/months: 5.9 +/- 0.7 vs. ≥2/months: 4.5 +/-0.2, p=0.002 | (3/4/13/7) | High |
| Murr 2007* [62] | **Hospital complications:** (aOR): ≥ 500 vs. 200-499: 0.9 (0.7–1.1); 100-199: 1.4 (1.1–1.6);  6-99: 2.0 (1.3–3.1) |  |  | (8/3/9/7) | High |

| **Hospital volume** | | | | | |
| --- | --- | --- | --- | --- | --- |
| **Author, year of publication** | **Morbidity:**  *Perioperative morbidity/ adverse events of the intervention/ complications*: including re-intervention and re-admission | **Morbidity:**  (long term-) Weight reduction; (long term-) BMI reduction; remission rate of comorbidities (diabetes mellitus, arterial hypertension, sleep apnoea, hyperlipidaemia, etc.) | **LOS** (Hospitals; ICU) | **Quality appraisal** | |
|  |  |  |  | **ISPOR**  (Yes/Partially/No/NA) | **ROBINS-E** (Domain 7†) |
| Bouchard 2020* [59] | **SG: 90-day major morbidity:** (aOR, 1 unit = 10 cases): 0.99 (0.97–1.01) \| **RYGB: 90-day major morbidity:** (aOR, 1 unit = 10 cases): 0.86 (0.77–0.96) {Included: complications: bleeding, venous thromboembolic event, pneumonia, macrovascular events (myocardial infarction and strokes), postoperative infection/leak, shock, need for de novo hemodialysis, reintubation, prolonged intubation (>48 hours), prolonged length of stay (LOS ≥7 days), or mortality} |  |  | (10/8/2/7) | Very High |
| Carbonell 2005 [43] |  | **Undefined morbidity:** (aOR): undefined volume: 0.94 (0.87 - 1.02); Morbidity: (percentages, adjusted multiple comparisons against all other groups): Very low vs. Other: 14.18%; Low vs. Other: 11.46%; Medium vs. Other: 11.65%; High vs. Other: 7.77%; p=0.0001 | **Hospitals:** (days) (percentages, adjusted multiple comparisons against all other groups): Very low vs. Other: 5.01; Low vs. Other: 4.87; Medium vs. Other: 4.41; High vs. Other: 2.88; p=0.0001 | (8/2/10/7) | Very High |
| Chadwick 2023* [19] |  |  | **Hospitals:** Average LOS: (adjusted beta regression weights, incremental DECREASE of volume):  SG: 0.155 days (0.132–0.178) days;  RYGB: 0.139 days (0.045-0.232) days; OAGB: Hospitals: NA;  LAGB: 0.262 days (0.218–0.307) days | (12/5/3/7) | Low |
| Chiu 2012* [60] |  |  | **Hospitals:** (days, adjusted beta hierarchical regression weights):  Low vs. High: -3.71 (-0.67), p<0.001 | (8/1/11/7) | High |
| Dimick 2009 [44] |  | **Risk-adjusted morbidity:** (aOR, 2005-2006):  Q1 (smallest) vs. Q4 (highest): 1.89 (1.47-2.43) |  | (7/3/10/7) | High |
| Doumouras 2017* [61] |  | **All cause morbidity:** (aOR): <200 vs. 200-400: 0.95 (0.73–1.20); >400: 1.70 (1.20–2.33) |  | (7/5/8/7) | High |
| Encinosa 2009 [45] | **Complications:** (180 days) (Log Odds): Low vs. Medium: 0.783 (0.013); High: 0.71 (0.003) (both significant (95% and 99%); \| **Complications:** post op hospital visits with complications: log odds:  Low vs. Medium: 0.84 (0.108); High: 0.706 (0.005) (high significant at 99% confidence level) |  |  | (9/4/7/7) | Low |
| Gould 2011 [46] | **Occurrence of one or more severe post- operative in-hospital complication and hospital mortality:** (aOR): >25 vs. 1-24: 1.13 (0.84–1.52); ≥50 vs. 1–49: 1.17 (0.94–1.45); ≥75 vs. 1–74: 1.21 (0.92–1.36): ≥100 vs. 1–99: 1.22 (1.01–1.49); ≥125 vs. 1–124: 1.31 (1.1–1.67) ; ≥150 vs. 1–149: 1.36 (1.1–1.67); ≥175 vs. 1–174: 1.57 (1.27–1.94); ≥200 vs. 1–199: 1.61 (1.29–2.01) |  |  | (11/2/7/7) | Very High |
| Hernandez-Boussard 2012 [15] | **Patient-Safety-Indicator:** (one or more, percentage): Low: 6.14%; Mid: 2.78%;High: 2.06%, p<0.0001 \| **Patient-Safety-Indicator** (risk adjusted, rate per 1000): **Pressure Ulcer:** Low: 15.12; Mid:14.2; High: 7.92, p<0.001 \| **Blood-stream Infection:** Low: 2.88; Mid: 0.74; High: 0.34, p<0.001 \| **Postoperative hemorrhage or gematoma:** Low: 2.93; Mid: 2.67; High: 1.69, p<0.0001 \| **Postoperative respiratory failure:** Low: 11.52; Mid: 9.6; High: 3.81, p<0.0001 \| **Postoperative pulmonary embolism/deep vein thrombosis:** Low: 14.35; Mid: 9.27; High: 7.87, p<0.0001 \| **Postoperative sepsis:** Low: 19.42; Mid: 16.05; High: 17.75, p=0.0875 \| **Postoperative wound dehiscence:** Low: 2.06; Mid: 1.66; High: 0.49, p<0.0001 \| **Accidental puncture or laceration:** Low: 4.4; Mid: 3.79; High: 3.36, p<0.0001 |  | **Hospitals:** (days) Low: 9.93; Mid: 10.10; High: 10.07, p=0.2096 | (5/3/12/7) | High |
| Hollenbeak 2008* [12] |  |  | **Hospitals:** (undefined): (GLM; identity link: Beta weight): High vs. Medium: 0.62, p<0.0001; Low: 1.42, p<0.0001 | (9/5/6/7) | Low |
| Ibrahim 2017 [47] | **Serious complication rate** (mean, sd, range %): Low: 1.9 (0.9) [0.6-6.4]; Medium: 2.0 (1.0) [0.5-10.3]; High: 1.5 (0.3) [0.6-4.9], p=0.31 |  |  | (7/10/9/1) | High |
| Jafari 2013 [48] | **Serious complication:** (aOR): High vs. Low: 1.2 (1.1-1.4) |  |  | (11/2/7/7) | High |
| Kauppila 2020 [14] | **Composite hospital mortality and reintervention:** (90 days, aHR):  Lowest vs. Medium: 0.7 (0.52 - 0.95); Highest: 0.8 (0.61 - 1.07) |  |  | (12/4/4/7) | High |
| Kelles 2009* [63] |  |  | **Hospitals:** (95%-ile, 5 days): (aOR): NA | (5/3/12/7) | High |
| Kohn 2010 [49] | **Any complication:** (aOR, incremental): 0.99937 (0.99888–0.99987) \| **Abdominal drainage:** 0.99869 (0.99752–0.99985 \| **Acute DVT:** 0.99989 (0.99859–1.00120) \| **Acute PE:** 0.99782 (0.99665–0.99898)\| **Myocardial infarction:** 0.99958 (0.99832–1.0008) \| **Cardiac complications:** 0.99873 (0.99782–0.99964) \| **Post-op shock:** 0.99776 (0.99591–0.99962) \| **Splenectomy:** 0.99813 (0.99682–0.99943) \| **Acute renal failure:** 0.99851 (0.99778–0.99923) \| **Acute CVA:** 1.00038 (0.99878–1.00199) \| **Bacterial pneumonia:** 0.99738 (0.99629–0.99848) \| **Respiratory failure:** 0.99896 (0.99806–0.99986) |  |  | (11/3/6/7) | High |
| Krell 2014 [50] | **Overall complication:** (Mean risk-and reliability adjusted rate, % (range)): Lowest: 7.6 (3.2-18.2); Medium: 6.8 (2.8-38.0); Highest: 5.5 (1.9-14.6) \| **Any serious complications:** Mean risk-and reliability adjusted rate, % (range): Low: 2.0 (0.9-9.6); Medium: 2.2 (1.2-8.6); High: 1.5 (0.5-7.0) \| **Reoperation:** Mean risk-and reliability adjusted rate, % (range): Low:1.0 (0.5-2.4); Medium: 0.9 (0.5-3.1); High: 0.9 (0.3-2.3) |  |  | (10/2/8/7) | Low |
| Liu 2003 [51] | **Serious complications:** (aOR): High vs. Medium: 1.3 (0.74 - 2.29); Low: 2.7 (1.41 - 5.2); Very low: 2.7 (1.57 - 4.73) |  |  | (8/3/9/7) | High |
| Murr 2007* [62] | **Hospital complications:** (aOR): ≥500 vs. 200-499: 0.9 (0.8–1.1); 100-199: 0.6 (0.4–0.7); 10-99: 1.3 (1.1–1.5); 1-9: 2.1 (1.2–3.5) |  |  | (8/3/9/7) | High |
| Nguyen 2004 [54] | **Overall complications**: (%): High: 10.2%, p<0.01 vs. Medium: 12.3%; Low: 14.5% \| **Pulmonary complications:** (%): High: 1.2%, p<0.01 vs. Medium: 2.0%; Low: 3.1% \| **Complications of medical care:** (%): High: 7.8%, p<0.01 vs. Medium: 9.5%; Low: 10.8% \| **Wound infection:** (%): High: 1.0%, p<0.01 vs. Medium: 1.2%; Low: 2.2% \| **Pneumonia:** (%): High: 0.7%, p insignificant, vs. Medium: 0.8%; Low: 0.8% \| **Venous thrombosis/pulmonary embolism:** (%): High: 0.3%, p insignificant vs. Medium: 0.3%; Low: 0.4% \| **Postprocedural hemorrhage:** (%): High: 1.3%, p insignificant vs. Medium: 1.4%; Low: 1.5% \| **Readmission:** (30 day, %): High: 0.3%, p<0.05 vs. Medium: 0.3%; Low: 0.6% |  | **Hospitals:** Mean LOS (days): High: 2.8 +/- 2.9, p<0.01 vs. Medium: 4.4 +/- 3.2; Low: 5.1 +/- 4.0 | (11/2/7/7) | High |
| Pradarelli 2016 [55] | **Postoperative complications:** (30 day, aOR): Low vs. Medium: 1.94 (0.89 - 4.23); High: 1.53 (0.71-3.28) |  |  | (9/5/6/7) | Low |
| Stenberg 2014 [56] | **Serious complication:** (aOR): >300 vs. 250-299: 1.17 (0.87-1.58); 200-249: 1.27 (0.97-1.66); 150-199: 1.47 (1.14-1.91); 100-149: 1.52 (1.14-2.02); <100: 1.83 (1.47-2.07) |  | **Hospitals:** (days, mean (sd)): >300 vs. 250-299: 2.2 (3.36), p<0.001; 200-249: 2.4 (3.17), p<0.001; 150-199: 2.3 (2.53), p<0.001; 100-149: 2.6 (2.23), p<0.001; <100: 3.0 (4.77), p<0.001 | (9/3/8/7) | Low |
| Svarts 2022 [16] | **Intraoperative complications:** (aOR) [1 unit =100 cases]:0.775 (0.69-0.871)  **30-day complications:** (aOR) [1 unit =100 cases]: 0.872 (0.806-0.943)  **One-year complications:** (aOR): [1 unit =100 cases]: 0.819 (0.707-0.949)'x+1.022 (1.006-1.039)^2'x; {quadratic linear index reported as beta'x+beta(CI)^2'x} | **% total weight loss after one year** (aOR) [1 unit =100 cases]: Volume:  0.00435 (0.0022-0.00651)'x+-0.000253 (-0.000439- -0.0000668)^2'x,{quadratic linear index reported as beta'x+beta^2'x} | **Hospitals:** (undefined, Incidence-rate-ratio): Volume: 0.881 (0.82-0.946) | (13/4/3/7) | High |
| Tsui 2020 [17] | **Revision to RYGB:** (percentages, CI): Low: 3.58% (2.56%, 4.59%); Medium: 4.28% (3.10%, 5.46%); High: 2.49% (1.64%, 3.35%), p=0.0221 \| **Hiatal hernia repair:** (percentages, CI): Low: 2.37% (1.53%, 3.22%); Medium: 1.48% (0.79%, 2.17%); High: 1.85% (1.10%, 2.60%), p=0.2822 \| **Other revision:** (percentages, CI): Low: 1.20% (0.65%, 1.76%); Medium: 1.23% (0.57%, 1.90%); High: 1.47% (0.82%, 2.12%), p=0.7098 |  |  | (8/1/11/7) | High |
| Varban 2015 [57] | **Re-operation:** (aOR, LAGB volume): 2006-2007: >125 vs. 50-125: 1.05 (0.63,1.76); <50: 1.44 (1.01,2.05)\| 2008-2009: >125 vs. 50-125: 1.23 (0.84,1.81); <50: 1.58 (1.12,2.25)\| 2010-2011: >125 vs. 50-125:0.78 (0.46,1.33); <50: 1.04 (0.67,1.63)  **Re-operation:** (aOR, LRYGB volume): 2006-2007: >125 vs. 50-125: 1.08 (0.83,1.39); <50: 1.27 (0.95,1.72)\| 2008-2009: >125 vs. 50-125: 1.12 (0.83,1.51); <50: 1.13 (0.82,1.56)\| 2010-2011: >125 vs. 50-125:1.12 (0.84,1.48); <50: 1.19 (0.88,1.61)  **Serious complications:** (aOR, LAGB volume):2006-2007: >125 vs. 50-125: 1.01 (0.66,1.55); <50: 1.65 (1.18,2.30)\| 2008-2009: >125 vs. 50-125: 1.27 (0.94,1.73); <50: 1.81 (1.36,2.41)\| 2010-2011: >125 vs. 50-125:1.16 (0.76,1.76); <50: 2.08 (1.40,3.09)  **Serious complications:** (aOR, LRYGB volume): 2006-2007: >125 vs. 50-125: 1.24 (1.00,1.54); <50: 1.55 (1.23,1.95)\| 2008-2009: >125 vs. 50-125:1.30 (1.05,1.61); <50: 1.39 (1.09,1.76)\| 2010-2011: >125 vs. 50-125:1.20 (0.96,1.50); <50: 1.39 (1.07,1.80) |  |  | (8/2/10/7) | High |
| Wilson 2015 [58] | **Readmission:** (aOR): Low vs. Medium: 0.92 (0.85–0.98); High: 0.79 (0.73–0.84) |  |  | (9/5/6/7) | Low |

Abbreviations: aHR - Adjusted hazard ratio; aOR - Adjusted odds ratio; aRR: Adjusted risk ratio; DVT - deep vein thrombosis; ISPOR: the international society for pharmacoeconomics and outcomes research; LOS - length of stay; LAGB - Laparoscopic adjustable gastric banding; NA - not applicable; OAGB - Omega-loop gastric bypass; PE - Pulmonary embolism; ROBINS-E - the risk of bias in non-randomized studies – of exposure; RYGB - Roux-en-Y gastric bypass; SG - sleeve gastrectomy; sd - standard deviation;

Note: *Studies that analyzed both hospital and surgeon volume appear twice in the tables. † Domain 7 = Risk of bias in selection of reported result.
